# Machine Learning Detects Anti-DENV Signatures in Antibody Repertoire Sequences

**DOI:** 10.3389/frai.2021.715462

**Published:** 2021-10-11

**Authors:** Alexander Horst, Erand Smakaj, Eriberto Noel Natali, Deniz Tosoni, Lmar Marie Babrak, Patrick Meier, Enkelejda Miho

**Affiliations:** ^1^ School of Life Sciences, Institute of Medical Engineering and Medical Informatics, University of Applied Sciences and Arts Northwestern Switzerland FHNW, Muttenz, Switzerland; ^2^ SIB Swiss Institute of Bioinformatics, Lausanne, Switzerland; ^3^ aiNET GmbH, Basel, Switzerland

**Keywords:** dengue, antibody repertoire analysis, machine learning, neural networks, long short-term memory networks, encoding, artificial intelligence, antibody discovery

## Abstract

Dengue infection is a global threat. As of today, there is no universal dengue fever treatment or vaccines unreservedly recommended by the World Health Organization. The investigation of the specific immune response to dengue virus would support antibody discovery as therapeutics for passive immunization and vaccine design. High-throughput sequencing enables the identification of the multitude of antibodies elicited in response to dengue infection at the sequence level. Artificial intelligence can mine the complex data generated and has the potential to uncover patterns in entire antibody repertoires and detect signatures distinctive of single virus-binding antibodies. However, these machine learning have not been harnessed to determine the immune response to dengue virus. In order to enable the application of machine learning, we have benchmarked existing methods for encoding biological and chemical knowledge as inputs and have investigated novel encoding techniques. We have applied different machine learning methods such as neural networks, random forests, and support vector machines and have investigated the parameter space to determine best performing algorithms for the detection and prediction of antibody patterns at the repertoire and antibody sequence levels in dengue-infected individuals. Our results show that immune response signatures to dengue are detectable both at the antibody repertoire and at the antibody sequence levels. By combining machine learning with phylogenies and network analysis, we generated novel sequences that present dengue-binding specific signatures. These results might aid further antibody discovery and support vaccine design.

## Introduction

### Large-Scale Sequencing Data Enables Machine Learning to Detect Patterns in Antibody Repertoires

In the field of bioinformatics, machine learning is broadly applied to a wide array of data such as electronic healthcare records and omics data to achieve a multitude of tasks such as disease classification or discovery and development of novel therapeutics. Lately, sequencing technologies have improved in terms of quality and costs declined by a factor of 50,000 resulting in the generation of tremendous amount of large-scale data (Goodwin et al., 2016). Applying machine learning (ML) to high-throughput sequencing (HTS) data can lead to a meaningful insight of the human biology. A subfield of HTS is focusing on unmasking the high complexity of the human adaptive immune receptor repertoire (AIRR) which can reach up to 10^12^ antibody clones. As the immune repertoire contains information about the past and current immune events such as infections and diseases, sequencing of such data could enable the prediction of health and diseases in patients and subsequently lead to the discovery of novel vaccines or therapeutics (Widrich et al., 2020). Even though the number of interactions within the AIRR is numerous, they can be described with a finite, universal vocabulary of interaction motifs (Akbar et al., 2021). Within AIRR sequences, each single amino acid is characterized by a series of biochemical/biophysical properties (e.g., hydrophobicity) which is recurrent in any antibody, even in sequences of antibodies with unrelated interactions. Therefore, ML can be used to extract patterns from sequence motifs in order to classify them. By studying the repertoire convergence, it is subsequently possible to generate new antibody candidates which can be used to develop new therapeutics and design novel vaccines (Greiff et al., 2015; Cinelli et al., 2017; Greiff et al., 2017). Research has successfully demonstrated the classification of diseased individuals by using deep neural networks on the immune repertoire data (Ibrahim et al., 2005; Hill et al., 2018; Widrich et al., 2020) but to our knowledge not on dengue repertoires.

### Dengue Virus is a Global Threat

The dengue virus (DENV) is a fever-causing virus of the Flaviviridae virus family classified into four different serotypes DENV I–IV (Muller et al., 2017). Additionally, a new serotype V was discovered recently with outbreaks restricted to Malaysia (Mustafa et al., 2014; Joob and Wiwanitkit, 2016). Dengue is a mosquito-transmitted disease often infecting an individual multiple times. While the primary infections are mostly asymptomatic or flu-like, the secondary infection can result in dengue haemorrhagic fever which can lead to death if it originated from another serotype than the primary infection. This happens because during the primary infection, antibodies are produced that lead to an exacerbation of the disease upon reinfection with a heterologous serotype (WHO, 2019; Parameswaran et al., 2013). Yearly, over 390 million cases are reported globally. Thereof, 500,000 patients need to be hospitalized with a mortality rate of approximately 2.5% (WHO, 2019). While five vaccines against dengue are still in clinical trials (WHO, 2020), a first vaccine has been developed: in 2019 FDA has approved a vaccine targeting previously infected patients. However, this vaccine is known to show efficacy only if the patient has had a primary infection and a secondary infection from a heterologous serotype and exhibits a more sever course of disease compared to previously uninfected patients (Godói et al., 2017). As of today, there is neither universal dengue fever treatment nor vaccines unreservedly recommended by the World Health Organization. Approximately half of the world’s population lives in dengue risk areas (Africa, Latin America, and Asia), and the disease is having an alarming impact on human health as well as global economies (WHO, 2019). Additionally, the lack of early-stage biomarkers makes it difficult to detect the dengue virus (Muller et al., 2017). Novel dengue diagnostics and treatments could have a beneficiary impact on both human well-being and economy. This and the promising results of machine learning-based analysis of HTS data have intensified efforts also in the field of dengue. Recently, scientists have started to sequence dengue and dengue-related antibody repertoires directly from human samples, leading to a tremendous amount of genomic data and an increased understanding of the genetic composition and diversity of the virus and its elicited antibodies (Parameswaran et al., 2013; Galson et al., 2014; Appanna et al., 2016; Huang et al., 2017).

### Machine Learning is Applied to the Dengue Data at the Antibody Repertoire and Sequence Level

HTS technologies generate an increased number and diversity of sequencing data compared to traditional methods (Goodwin et al., 2016). Therefore, using machine learning to analyze HTS data might lead to impactful discoveries of rare and novel broadly neutralizing dengue antibodies (bNAbs) against all four serotypes that could serve as antibody therapeutics for passive immunization. In addition, the identification of DENV antibody repertoire signatures is an important milestone toward vaccine design and commercial development.

Our findings contribute to the discovery of DENV bNAbs by investigating different amino acid encoding methods and introducing a novel, **physicochemical** property-based encoding strategy, and benchmarking various machine learning methods to predict dengue progression and dengue-specific antibodies from high-throughput sequences of antibody repertoires.

## Materials and Methods

### Data

**Dataset 1:** Parameswaran et al. (2013) analyzed dengue antibody heavy-chain IgG signatures in 60 acute, post-recovery and healthy samples from Nicaragua containing 1) 44 samples with DENV in different phases: infection-associated signatures (acute), persistence of signatures post-clearance of infection (convalescent), and baseline profiles (pconv) within the same individual; 2) eight samples with non-dengue illnesses (non-dengue); and 3) eight healthy samples (healthy). Sequencing was performed twice by independent GS FLX (454 Life Sciences/Roche) runs.

**Dataset 2:** Godoy-Lozano et al. (2016) analyzed dengue antibody heavy-chain IgG signatures of 19 acute (den_A) samples from Mexico. Six months later, 11 post-convalescence samples were taken (den_PC). All samples are available as BioProject with ID PRJNA302665. Sequencing was performed using a Roche 454 sequencer.

**Dataset 3:** Huang et al. (2017) analyzed dengue antibody heavy-chain IgG/IgA signatures of 14 hemorrhagic, simple and healthy samples from Taiwan available as BioProject with ID PRJEB13768 (IgG and IgA antibodies). Since several repertoires were taken from each patient throughout the progress of the disease, a total of 59 repertoires were available. Sequencing was performed as 150 bp paired-end sequencing using an Illumina NextSeq machine.

**Dataset 4:** While datasets 1 through 3 provide mostly signatures found in dengue-challenged repertoires, additional sequences from healthy patients were collected from iReceptor, a public repository of sequencing data. The selection criteria were set accordingly so that only heavy chain, productive sequences of healthy samples were retrieved. In total, approximately 62 million sequences were downloaded with all of them being annotated with IMGT blast.

### Annotation and Pre-processing

Reads were labeled according to the dataset. In dataset 1 reads were labeled either as acute, convalescent, postconvalescent (p-convalescent), healthy, or non-dengue. In dataset 2 reads were labeled as acute or p-convalescent. Dataset 3 distinguishes between hemorrhagic dengue, simple dengue, and healthy. To distinguish sequences of dengue infected individuals from healthy individuals, we mapped the original 21 classes into two new classes: dengue-challenged and non-dengue-challenged that can be considered as a binary classification problem (Supplementary Figure 1). Raw sequences across datasets were annotated with IgBLAST. CDR3 sequences were filtered for productivity, minimum CDR3 sequence length of four amino acids, and only sequences which were present more than once were retained for further analysis in order to filter out potential spurious results from sequencing errors (Smakaj et al., 2019). After performing initial filtering, a total of 2.7 million sequences were analyzed and pulled into one dataset used for benchmarking the encoding methods and training of the machine learning models.

### Encoding

Encoding refers to the process of transforming text or sequence data into numeric data which can be input to a machine learning algorithm. The same input data were represented differently in order to select among different encoding methods and, therefore, each encoded input had a variable impact on machine learning measures. In computational biology, encoding of amino acids can be achieved by considering amino acids’ physicochemical *properties*, for instance, using the BLOSUM substitution matrix, or by a generic *character-wise encoding* like one-hot or integer encoding used also in other ML domains (Zamani and Kremer, 2011).

**FIGURE 1:**
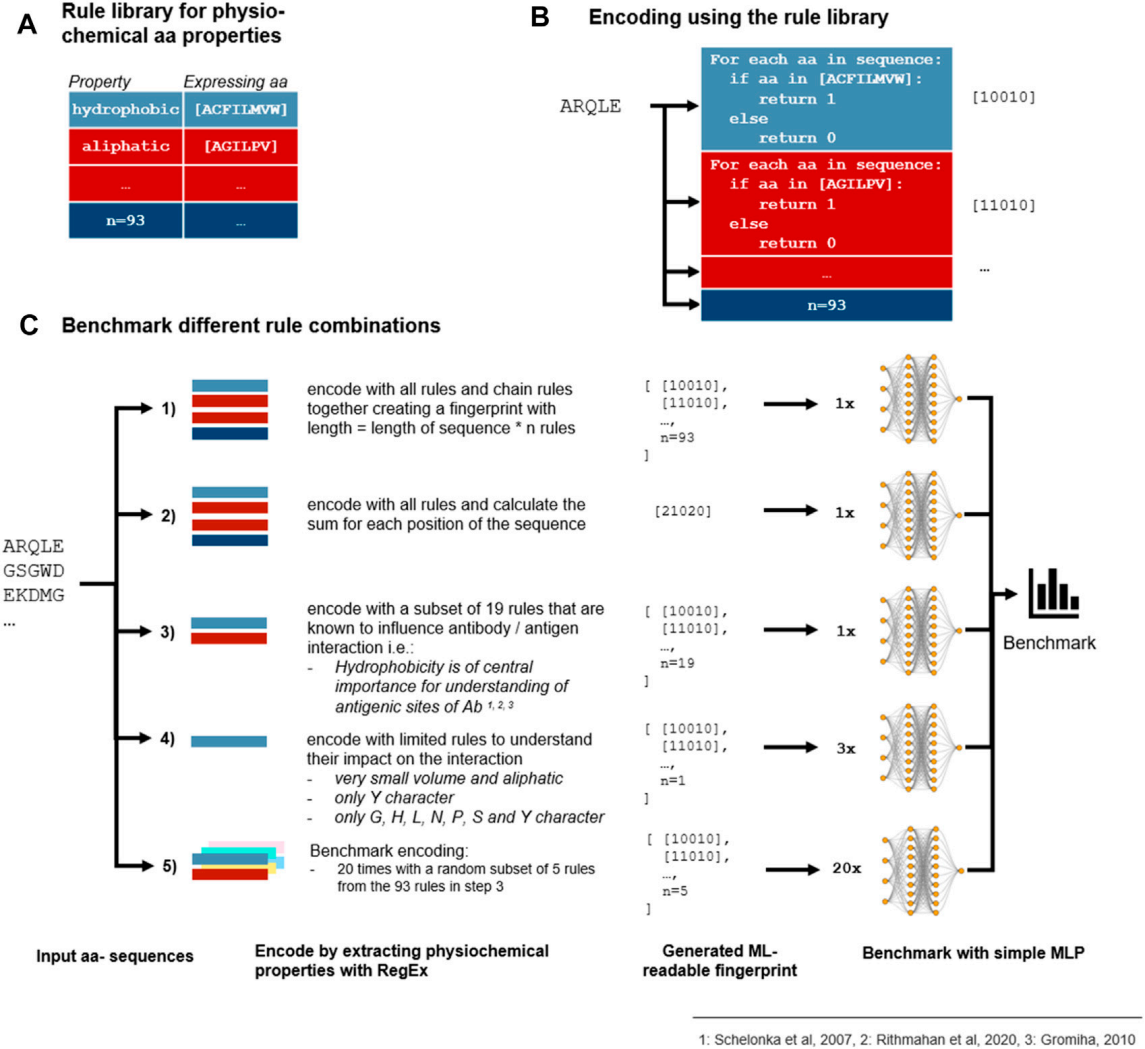
Benchmark of encoding with different physicochemical rules. **(A)** 93 rules were defined that describe the physicochemical properties of an Ab which are expressed as different amino acids in a CDR3 sequence. **(B)** Each rule is represented by 1’s and 0’s which are extracted from the CDR3 a.a. sequence by using a regular expression (RegEx). **(C)** As the rules can be combined differently, we benchmarked different encoding methods which all depend on the rule library to find the best combination of rules. First, every numeric representation of the rules is chained together (Goodwin et al., 2016) or the sum for each a.a. position is calculated (Widrich et al., 2020). Second, as some rules are repeated because some properties are expressed in the same manner and some physicochemical properties are known to influence the antibody–antigen interaction more than others, a subset of 19 rules was selected (Akbar et al., 2021). Additionally, a randomly selected subset of 5 rules out of the 19 rules from step 3 was combined with 3 rules to analyze the contribution of single properties to the prediction results. As shown in the fivefold validated benchmark, chaining of the selected subset outperforms chaining and summing of all rules. As no subset of five rules was able to contend with more extensive encodings, we come to the conclusion that not a few single properties but the combination of various properties is accountable for proper sequence classification.

In addition to taking into account the existing encoding schemes indicated in Table 1, we additionally introduced a novel encoding scheme where the encoding was based on each amino acid within the CDR3 sequence. Each amino acid represents different physicochemical properties, for instance, amino acid A (alanine) represents the property aliphatic; therefore, the compound contains carbon and hydrogen which make up an aliphatic functional group on the side chain (Schelonka et al., 2007; Ritmahan et al., 2020). We compiled this information in a rule library (Figure 1A) which enabled the comparison of each amino acid within a given CDR3 sequence against the library (Figure 1B). We aimed to further improve the results by combining the rules for those properties which were shown to have the highest impact on the antibody–antigen interaction (Figure 1C; Supplementary Appendix S1 for all rules). By random subsampling of five rules from the rule library, additional insights on which rules are most contributing to favourable classification results shall be obtained (Figure 1C).

**TABLE 1:** Seven encoding methods were benchmarked for their suitability to represent CDR3 a.a. sequences.

Encoding	Type	Explanation
One-hot	Numeric encoding	Each amino acid (A to Y) is represented by a binary vector leading to a total of 20 vectors with a length equal to the longest CDR3 sequence in the dataset. Each position in the vector represents the amino acid at that position within the sequence. If the amino acid at a specific position equals to the amino acid represented by the vector, the position becomes 1 otherwise 0. An CDR3 sequence with a length of three would therefore be represented, by twenty binary vectors, each of length two.
Integer encoding	Numeric encoding	Each amino acid is mapped to a number such that a number represents always the same amino acid character. An amino acid sequence with length three would therefore be represented, by one integer vector of length three.
k-mers	Property based	Each amino acid sequence is split into several smaller subsequences of length k. Each subsequence is then treated as a token which is compared to other tokens found (Zamani and Kremer, 2011; Hill et al., 2018). By then applying term frequency-inverse document frequency (or any other information retrieval algorithm) a numeric weight is applied to each token.
BLOSUM50/62/80	Property based	Sequence alignment using the BLOSUM50, BLOSUM62 and BLOSUM80 substitution matrix (Henikoff and Henikoff, 1992). BLOSUM with high numbers are used for highly related proteins while lower numbers are used for more distantly related proteins. Although BLOSUM 62 was proven to be miscalculated and therefore not being precisely accurate, it still delivers high performance results explaining why it is broadly used in protein sequence alignment and encoding (Styczynski et al., 2008).
Physicochemical rules, chained	Property based	Sequence alignment using a set of physicochemical rules (Figure 3, Supplementary Appendix S1). The rules are chained back-to-back together creating a numeric fingerprint per CDR3 sequence.
Subset of physicochemical rules, chained	Property based	Research has shown, that some physicochemical properties, for instance hydrophobicity, contribute stronger to the antibodies ability to bind to an antigen (Schelonka et al., 2007; González-Muñoz et al., 2012; Laffy et al., 2017; Ritmahan et al., 2020). Therefore, a subset of rules was chosen according to the estimated binding contribution (Figure 3, Supplementary Appendix S1).
Physicochemical rules, summed	Property based	Sequence alignment using a set of physicochemical rules (Figure 3, Supplementary Appendix S1). The rules then are then column-wise summed together.

### Machine Learning Models

After determining the best suitable encoding schema, the different models were trained with the labeled CDR3 sequences encoded accordingly. To do so, 80% of the data was randomly assigned to a train and validation set while 20% of the data was kept aside as a test set. To prevent the models from overfitting, training was performed using k-fold cross-validation with k = 5. This means, the train and validation data were split into five partitions and the models were trained five times, using every partition once as a validation set and then taking the mean measure as the final measure. With this procedure, the measures do not reflect results of only one validation set and chances of overfitting were lowered. Cross-validation was applied among all machine learning models: multilayer perceptron (MLP), recurrent neural network (RNN), long-short term neural network (LSTM), random forest, and support vector machine (SVM) algorithms which are shortly introduced below.

**FIGURE 2:**
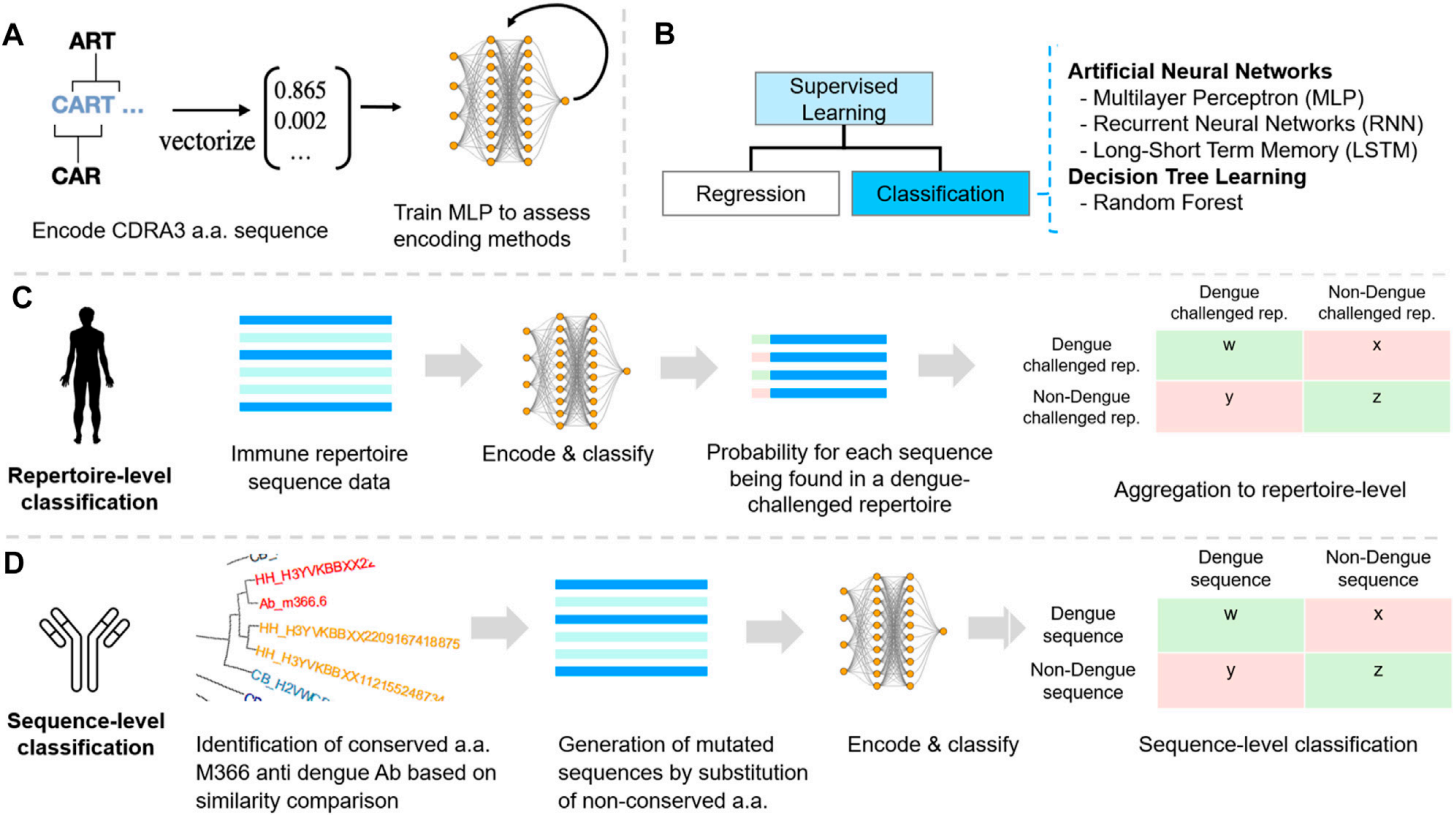
Machine learning reveals dengue-specific antibody sequence signatures at the repertoire and sequence level. **(A)** Encoding. Five different encoding schemes were benchmarked using a simple multilayer perceptron (MLP). **(B)** Classification of CDR3 sequences was performed using supervised machine learning classification as for each repertoire and sequence the label (dengue/non-dengue) was known. **(C)** Dengue datasets were annotated using IgBAST and preprocessed in R (see Methods). Labelled (dengue/background) CDR3 sequences were subsequently encoded. ML models were tuned, trained, and compared with regard to accuracy, sensitivity, specificity, and efficiency at the repertoire-level and sequence-level classification. **(D)** Sequence classification was performed based on bNAb sequences and constructing *de novo* bNAb networks to detect extended sequence-patterns in repertoires. Benchmarking various encoding methods.

Artificial neural networks (ANNs) are a supervised learning algorithm (Figure 2B) that recognize and learn patterns in data that are often not visible to the human eye. The algorithm then applies these patterns to new data and is thus able to make forecasts and predictions (Hansen and Salamon, 1990). Although, there are a large variety of different ANN architectures, this research focuses on three architectures. The multilayer perceptron (MLP) was chosen as it is often used as a baseline model to compare more complex models again. The recurrent neural networks (RNNs) and the long-short term memory network (LSTM) have been chosen because of previous success in predicting protein binding and secondary structure (Sønderby et al., 2015; Lipton et al., 2017).

Deep feed forward (DFF) neural networks are used to predict the progression of dengue infection from antibody repertoires. In order to avoid bias in the training data, the labels and the classes were balanced by upsampling the data using the caret R package (function upSample). Upsampling here means that we have sampled with replacement from the subset which contains fewer data points in order to obtain an equal amount of training data to the other classes (Table 1).

Quantifying statistical data from texts is necessary in order to extrapolate text into numbers and subsequently apply machine learning in a numeric representation of the data. For this purpose, the CDR3 amino acid sequences were further transformed into series of trigrams (series of 3 consecutive letters from a string, e.g., trigrams of the string “example” are “CAR, TAR, KLE, ERA, and GIT”) and the resulting vectors were transformed into tensors using the tf-idf function.

tf-idf (term frequency * inverse document frequency) is a numerical statistic of word occurrences in a given body of texts. In our case, the body of texts is the whole data, a document is an individual sequence and a word is an individual trigram. The list of all possible trigrams is called a dictionary. tf-idf computes the frequency of the word in a dictionary then multiplies it by the frequency of the document in the body of texts. This numerical representation is preferred over other methods of quantifying text frequency because it scales the occurrence frequency of an individual word (in our case a trigram) based on the occurrence frequency of the document (in our case a.a. sequence) in which the word is found.

By combining the dictionary with tf-idf for each trigram, it is possible to obtain a numerical trainable matrix representing the whole data. Finally, to obtain the training tensors, each tuple (sequence + class label) is defined as a dictionary matrix where each row corresponding to trigrams not available in the original sequence has been multiplied by 0. This gives a sparse matrix of tf-idf values present only in the row coordinates of trigrams in the sequence in question. Since the number of rows in this sparse matrix is equal to the number of terms in the dictionary, the resulting training tensor is very large in one dimension. For instance, a dictionary of 3-grams created from amino acid sequences has a theoretical maximum term count of W = 20^n (*n* = 3 for trigrams, W = 8,000, in general form this method allows to vary n by reproducing with another type of n-gram). In order to achieve realistic training times, we have reduced the dictionary size to 2048 top terms by frequency. We have justified this value heuristically on a 20/80 rule applied to term frequencies in the dictionary as a whole.

We proceed in using these tensors in training a keras deep learning network to classify each CDR3 with one of the given labels using a sequential model. The model consisted of a DFF architecture with rectifier linear unit (ReLU) activation function. The network’s input structure was generated depending on the vocabulary size (in this case 2048x1 as described above). The overall network structure used was (W x ReLU) + (W/2 x ReLU) + (10 x ReLU) + (5 x softmax). Once the model was trained, it predicted the class of each sequence of a given repertoire. The repertoire label was assigned based on a majority vote for the sequence labels it contained.

For random forests (RF), multiple, uncorrelated decision trees (called single predictors) were built. Each tree was grown on a random subset of the training data. The features used for each tree were a random subset of features contained in the training data subset. This randomization led to a forest of trees with different shapes and depths. For classifying a new sample, each single predictor was run through and the resulting class of each predictor was counted. The class with the most votes was considered the final prediction (Breiman, 1999; Livingston, 2005; Polaka et al., 2010).

Support vector machines (SVMs) are used to predict bNAb-like CDR3 sequences. We discriminated dengue bNAb-like (based on a database of 26 bNAbs) versus non-bNAb-like CDR3 clones based on the CDR3 sequence using the KeBABS R package (Palme et al., 2015). In brief, KeBABS enables the kernel-based analysis of biological sequences using a position-independent gappy pair kernel that divides sequences into features of length k with gaps up to length m. For example, the sequence CARTA is decomposed by the gappy pair kernel with parameters k = 1 and m = 2 into monomers with gaps of zero to two amino acids in between: CA, C.R, C.T, AR, A.T, A.A, RT, R.A, and TA. We first calculated the balanced accuracy of the two classes of sequences versus each other as previously described (Greiff et al., 2017; Miho, 2017): dengue-challenged and non-dengue-challenged. We built an SVM model from equilibrating the input sequences for the classes. We trained the classifier by setting 80% of sequences as a training dataset and 20% of the sequences as a test dataset.

### Tuning Machine Learning Models

The ANN are hypertuned on the parameters shown in Supplementary Table 1S with the R package keras and the random forest is tuned on the parameters shown in Supplementary Table 2S with the R package Random Forest.

SVM parameters were set to k = 3, m = 1, and C = 1 (C is the cost for the misclassification of a sequence) after searching the parameter space for the optimal model by nested cross-validation. The prediction accuracy of class discrimination was quantified by calculating the balanced accuracy (0.5 * (Specificity + Sensitivity)), where specificity was defined as TN/(TN + FP) and sensitivity as TP/(TP + FN) with TP, TN, FP, and FN being true positive, true negative, false positive, and false negative, respectively.

For all classification methods, the receiver operating characteristic (ROC) curve and the thereof derived area under the curve (AUC), which are reliable performance meassures, were calculated are reliable performance measures are were calculated. The ROC is a probability curve where the true positive rate (sensitivity) is plotted as a function of the false positive rate (100-Specificity) for each possible data point. A high discrimination (limited overlap in two distributions) has a ROC curve close to the upper left corner (Zweig and Campbell, 1993).

### Dengue Antibody Repertoire and Antibody Sequence Classification

After selecting the appropriate encoding and machine learning methods, the models were trained to classify repertoires of individuals as dengue-challenged or non-dengue-challenged based on a cross-repertoire signature found by training on multiple repertoire data. Therefore, for each sequence within the repertoire, the probability of being a dengue sequence was calculated. If the mean probabilities per repertoire were above a threshold of 0.5, the repertoire was assumed to be dengue-challenged. Furthermore, the models were trained on dengue-specific CDR3 sequences reported by Xu et al. (2017a) and Hu et al. (2019a). In order to increase the number of dengue-specific sequences, random mutations were computationally introduced to dengue-specific sequences. Specifically, the germlines were analyzed in order to determine conserved positions by means of phylogenetic trees. Each non-conserved position was subsequently randomly substituted by any other amino acid creating additional sequences which could be used for training the models on a sequence-level.

### Similarity Networks

Networks were constructed with each CDR3 amino acid sequence representing a node linked to its most similar sequences with the Levenshtein distance (LD) = 1, edit of one amino acid.

## Results

### Machine Learning can Classify Dengue-Challenged Antibody Repertoire Sequences

We used machine learning to classify sequencing data of dengue-challenged antibody repertoires (Figure 2). We investigated different amino acid encoding methods and introduced a novel, physicochemical property-based, encoding strategy (Figure 2; Supplementary Figure 1S). We tested the feasibility of applying machine learning to classify dengue high-throughput antibody repertoire sequences by testing a neural network model to classify repertoires of dengue stages (Supplementary Figure 2S). Various ML methods were applied to multiple dengue antibody repertoire sequencing datasets in order to identify sequence patterns within the complementarity-determining region 3 (CDR3) representing a DENV-specific signature on the antibody repertoire and sequence levels. The methods enabled a repertoire-level classification of dengue progression and identified dengue-challenged versus non-dengue repertoires. We focused on the CDR3 region because this is the major site of antigen recognition and therefore represents the most attractive target for sequence-based antibody specificity predictions (Thakkar and Bailey-Kellogg, 2019). Additionally, ML was applied at the antibody sequence level to distinguish between dengue and non-dengue sequences within a dengue-challenged repertoire. By training ML methods with DENV bNAbs sequences, we detected intrinsic sequence features that allow uncovering sequence-associated signatures of dengue-specific antibodies.

**FIGURE 3:**
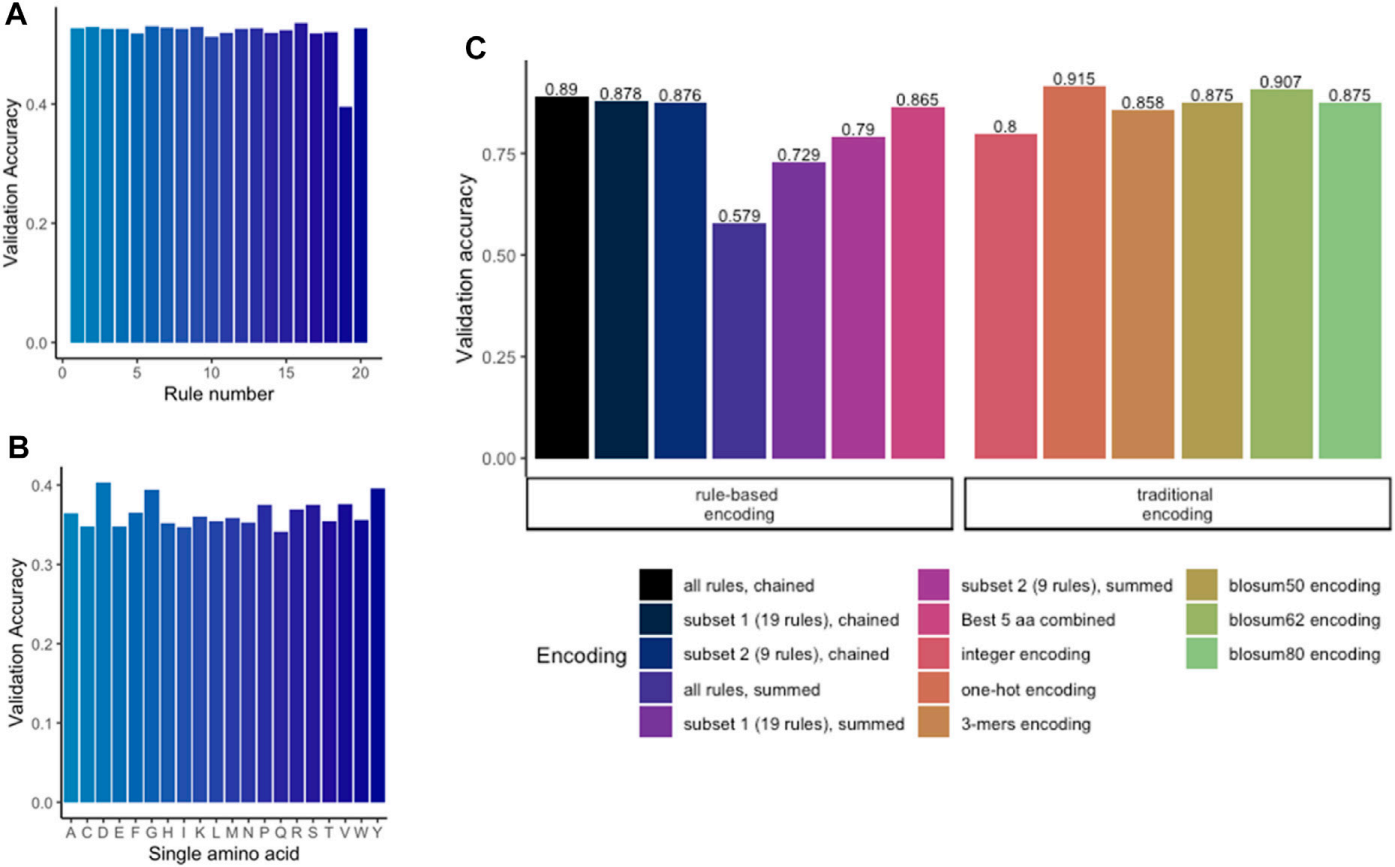
Benchmarking of encoding methods. **(A)** Validation accuracy for random chaining of five physicochemical rules. **(B)** Validation accuracy for encoding with only one single amino acid per model. **(C)** Comparing the rule-based methods with traditional methods revealed that one-hot encoding achieved the highest accuracy. Highest accuracy among the rule-based encoding is achieved by chaining the subset of 19 rules.

To apply any ML methods to dengue sequencing data, we investigated various encoding methods (Table 2; Figure 1). To evaluate different encoding methods, the data were encoded with each method (Table 2) and then fed into an MLP network. First, the rule-based encoding methods were benchmarked. To establish a benchmarking baseline, 20 * 5 random rules were selected from the library resulting in validation accuracies shown in Figure 3A. Then, for a better understanding of which amino acid accounted for high accuracy, only one amino acid was encoded as 1 while other amino acids were encoded as 0 leading to 20 different trainings of the model as shown in Figure 3B. Results indicated that amino acids D, G, P, R, S, V, and Y alone have the highest accuracy rate. However, a combination of these amino acids (Figure 3C, black bar) was still outperformed by larger signature sizes as indicated in Figure 3C. Figure 3D shows validation accuracy for integer, one-hot, k-mers, physicochemical rules-based, and BLOSUM/62/80 encoding. For a balanced dataset (equal number of samples in all classes) with two classes, random guessing would lead to approximately 50% accuracy. Therefore, the benchmarked encoding methods need to achieve more than 50% accuracy to perform better than random guessing.

**TABLE 2:** Accuracy, sensitivity and specificity, and AUC and training time.

Model	Accuracy (%)	Sensitivity (%)	Specificity (%)	AUC (%)
MLP	95.62	98.51	92.73	98.65
RNN	96.42	97.25	95.54	98.93
LSTM	96.67	97.77	95.56	98.99
Random Forests	92.66	95.08	90.25	96.04

On the one hand, methods which do not preserve any information about the sequence order like the physicochemical rules-based summation or the integer encoding, tended to achieve lower accuracy measures. These procedures consider only the position of a single a.a., but not which a.a. is found up- or downstream in the sequence. On the other hand, encoding methods which do preserve the sequence order achieved a higher accuracy. One-hot encoding achieved the highest accuracy (more than 91% balance accuracy), followed by BLOSUM62 and chaining of physicochemical rules. All BLOSUM encodings obtained a score of ≈90%. k-mers, which also preservers the sequence order, achieved an accuracy of 85.6% but started to overfit after five epochs (data not shown). k-mers could potentially be further improved by expanding the key size. Nonetheless, this was not further assessed as the one-hot encoding offered the highest and sufficient accuracy to be used for further training.

### Comparison of the Performance of Different Machine Learning Architectures

**FIGURE 4:**
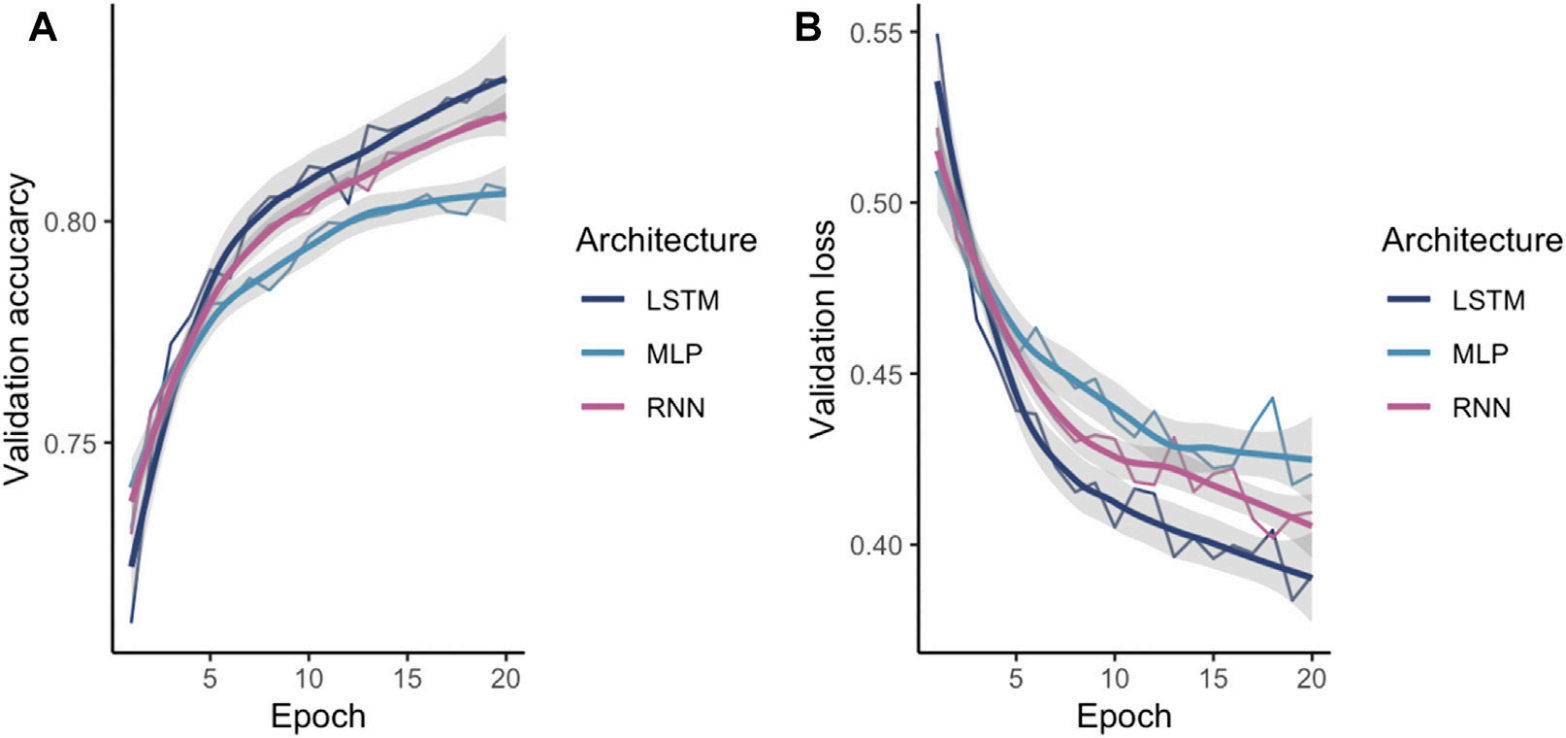
Comparison of the validated NN models **(A)** accuracy and **(B)** loss. The X-axis shows the number of epochs while the Y-axis shows the validation accuracy or the validation loss respectively.

After identifying one-hot as the encoding method, the encoded data were fed into different ML models in order to investigate the best suited architecture and parameter set. The tuned ANN validation accuracy and validation loss during training is shown in Figure 4. As expected, LSTM and RNN both outperformed a more naïve MLP as these models are aware of the regions upstream in the sequence.

**FIGURE 5:**
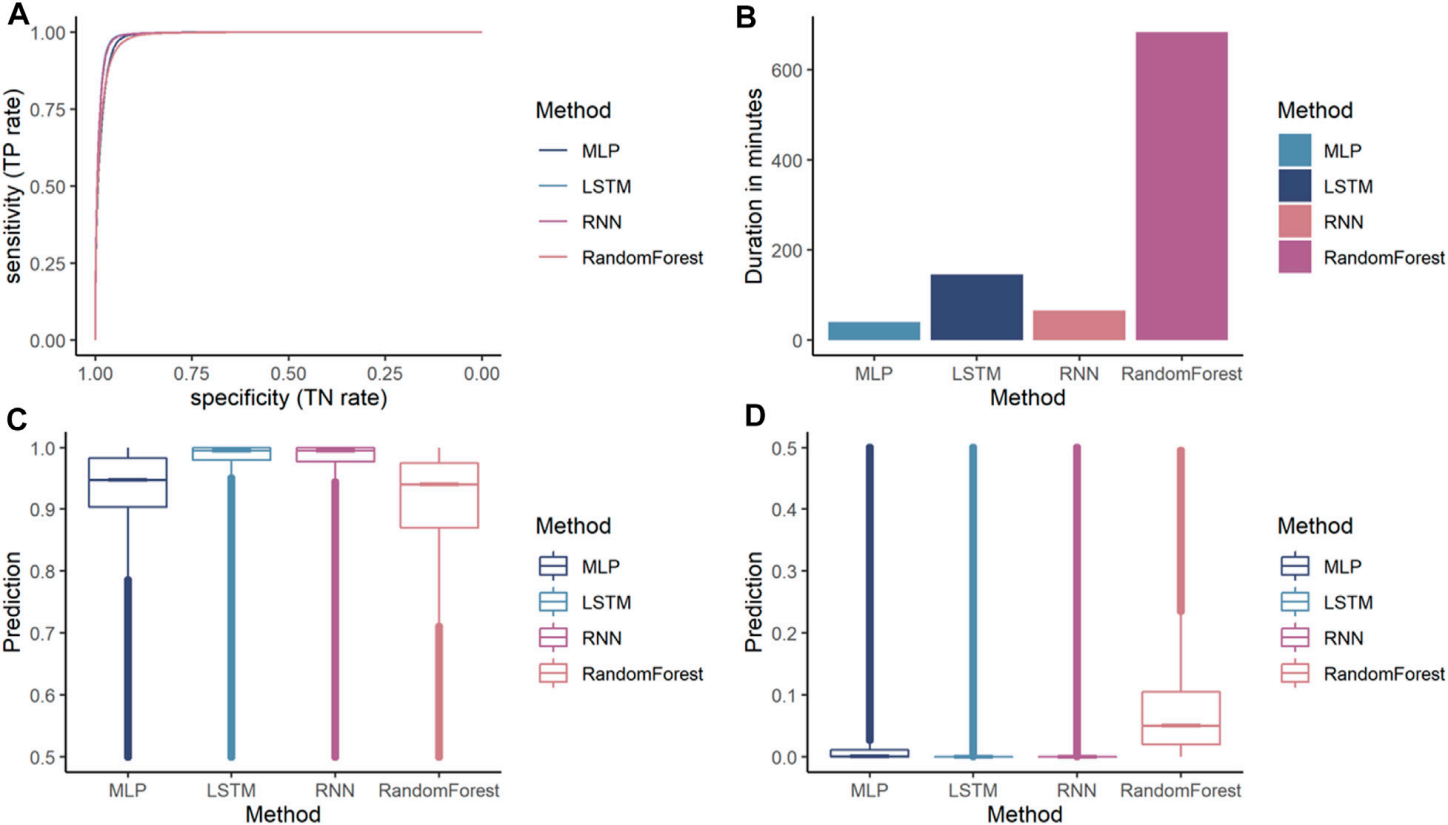
Comparison of different machine learning models on dengue CDR3 sequences. **(A)** Receiver Operating Characteristic (ROC) curves for MLP, RNN, LSTM, and random forest provides a specificity based on the sensitivity. The X-axis shows the specificity in function of the sensitivity (100-Sensitivity, false-positive rate) while the Y-axis shows the sensitivity (true positive rate). **(B)** Training times of one k-fold (Y-axis) for the given ML models (X-axis) indicating that random forest is by far the most computationally intense models. MLP and RNN are roughly the same while LSTM needs approximately twice as long compared to the other ANN models. **(C)** Median, confidence intervals, first and third quartile, and min and max values for the positive class (dengue). This indicates that the confidence for most dengue predictions is highest for the LSTM model, followed by the other ANN and random forest predicting with lowest confidence. **(D)** Median, confidence intervals, first and third quartile, min and max values for the negative class (background). This indicates that LSTM and RNN are the most confident in predicting the background class followed by the MLP.

Additionally, a random forest was trained. While all models achieved an accuracy over 90%, the MLP is the lowest performing ANN achieving an accuracy of 95.62%. RNN and LSTM achieved similar accuracies 96.42 and 96.67%, respectively. The random forest performs lower compared to the ANN achieving 92.66% accuracy. All models have a higher specificity than sensitivity, therefore these models were more accurate in predicting the negative class as negative (i.e., background sequence as background). However, a higher sensitivity is for this prediction favorable as it is more relevant to correctly predict the disease than predicting the background. The area below the ROC, referred to as AUC, represented how well the models could distinguish between two classes (Table 2 for AUC and Figure 5A for ROC).

Random forest is by far the most computationally expensive model regarding training times (Figure 5B). It needed 684 min to train a single fold making it more than four times slower than that of the other models. The RNN and the MLP were equally efficient and needed approximately 90% less time making them the most efficient models in the comparison. The LSTM needed only 21% of the time compared to random forest making it the most inefficient out of the benchmarked ANN models.

While accuracy and ROC showed how accurately the models performed the prediction, they did not indicate how confident the model was in its prediction. For our binary classification purpose, a probability of 100 and 52% return the same predicted class as both are above the threshold 50. However, a prediction probability of 100% would be preferred, indicating that the model is more confident in its prediction. The prediction confidence of each model for the positive class (dengue) is shown in Figure 5C, and the prediction confidence for each model for the negative class (background) is shown in Figure 5D. ANNs are more confident with their positive and negative predictions compared to the random forest model.

From the ANNs, the simple MLP was the most inaccurate while also being faster during training than the LSTM and the RNN models. The LSTM model has a slightly higher accuracy while the training times are considerably slower than the proposed MLP and RNN architecture. Even though, the random forest achieves accuracy measures close to the ANN models the ANN tend to make more confident predictions. Therefore, LSTM seems to be the most suitable architecture for the given classification task.

### Antibody Repertoire-Level Dengue Classification

**FIGURE 6:**
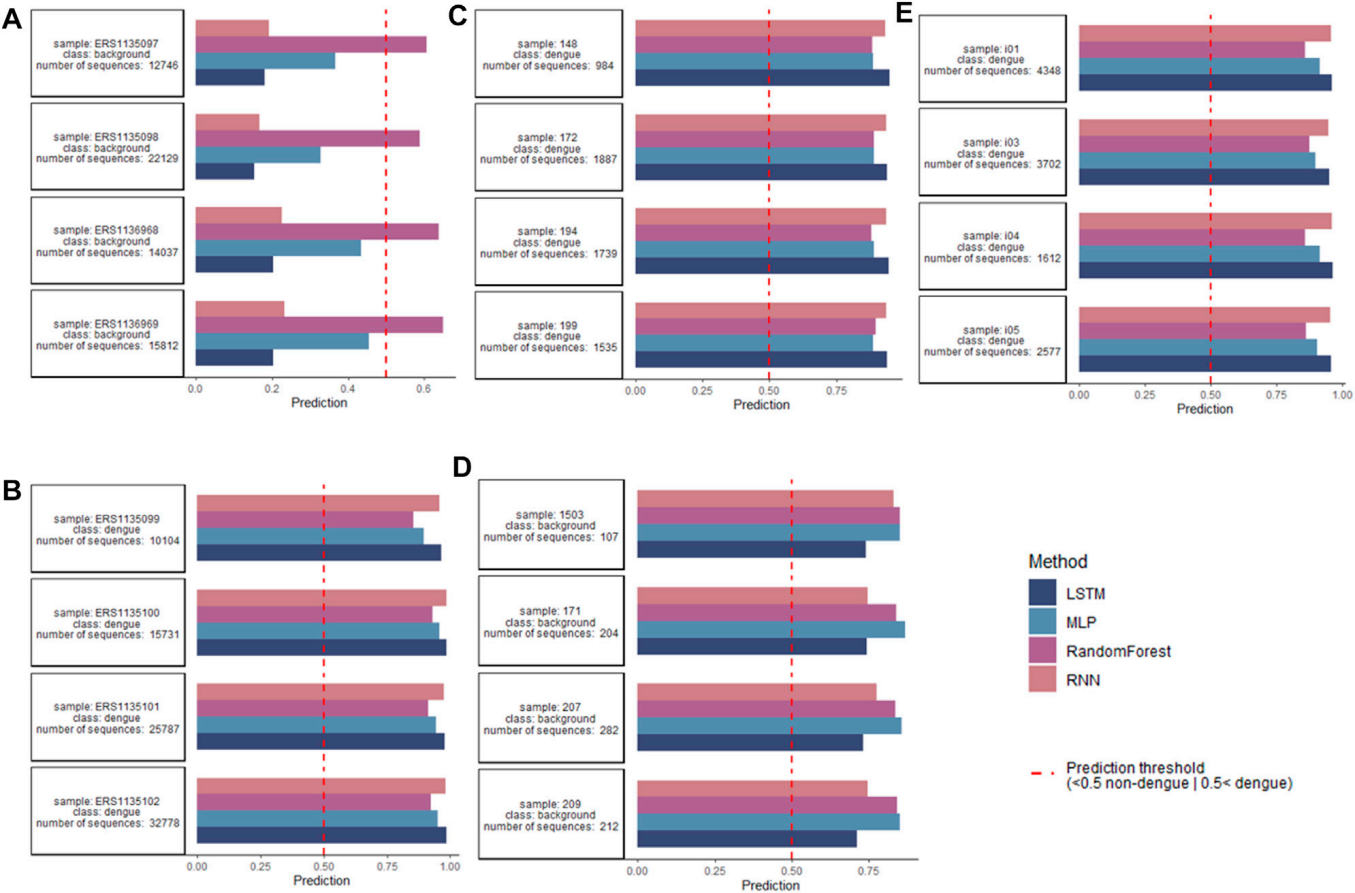
Classification on repertoire level per used dataset, for readability only four repertoires per class. **(A,D)** Classification performed for non-dengue-challenged repertoires found in Parameswaran et al. (2013) and Hwang et al. (2018). While in **(A)** all repertoires were classified correctly by the ANN models, in **(D)** all repertoires were mistakenly classified as dengue-challenged, likely due to a low number of sequences (<1,000) within the repertoires. **(B,C,E)** Classification performed for dengue-challenged repertoires found in Parameswaran et al. (2013), Godoy-Lozano et al. (2016); and Hwang et al. (2018) where all repertoires were correctly classified as dengue-challenged. Sequence-level basis for classification of dengue repertoires and antibodies.

After encoding data with one-hot, the models were trained to classify repertoires of individuals as dengue-challenged or non-dengue-challenged based on a cross-repertoire signature found by training on multiple repertoire data. Figure 6 indicates the repertoire-wise classification per original dataset for non-dengue-challenged (A and D) and dengue-challenged repertoires (B, C, and E). In total 118 dengue-challenged repertoires and 19 dengue-challenged repertoires were classified, both classes with ≈125,000 sequences each. From the 118 dengue-challenged repertoires, all were classified correctly regardless of the model while from the 19 non-dengue-challenged repertoires only 5 were classified correctly by the ANNs and 1 by the random forest. The 5 correctly classified repertoires contain more than 12,000 sequences each, while those repertoires that were misclassified as dengue-challenged are very small, each consisting of less than 550 sequences; this might be a potential explanation for the misclassification. If we take into consideration only the 93 repertoires with more than 1,000 sequences, all repertoires were classified correctly (5 as non-dengue and 88 as dengue).

In order to understand the misclassification, we further investigated the repertoires at the sequence level (Supplementary Figure 3S). By representing the CDR3 amino acid sequence in a similarity network (Miho et al., 2019), the ML predictions could be better understood. Adding the ML predicted and the actual class to each node of the network allowed us to observe if the model classifies similar sequences equally. Comparing misclassified sequences in the background repertoires with the similarity network indicated that these sequences are highly similar to sequences found in dengue-challenged repertoires and therefore, classified as dengue sequences. For the majority of the similarity networks (14 out of 15 groups), ML classifies all similar sequences equally as either background or dengue-challenged. For some similarity networks (i.e., 52, 431, and 1,359) ML classifies all sequences as background sequences although roughly 10% are dengue sequences. During the data prepossessing stage, all sequences found within a dengue-challenged repertoire were labeled as dengue-related. However, this simplification does not reflect the biology of the repertoire, as not all CDR3 amino acid sequences present in dengue patients are dengue specific but rather could be the result of any other non-dengue infection or background antibodies and can be considered as a training error. ML classifies these similar sequences together as background sequences, leading to the assumption that the ML classification corrects the simplification leading to a training error.

**FIGURE 7:**
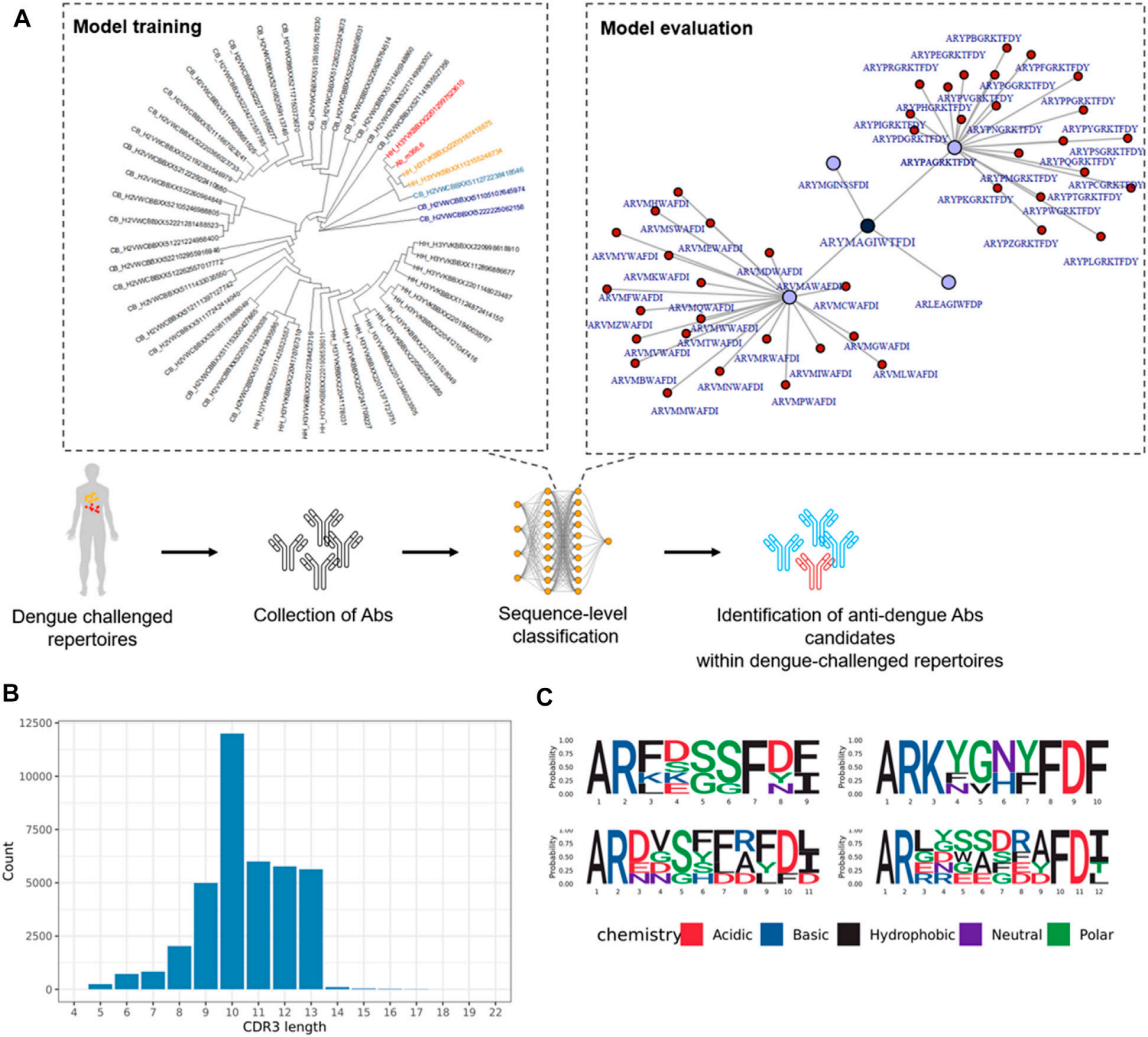
Dengue-specific antibody sequences generated with network analysis and machine learning. **(A)** Sequences with highest similarity to Ab m366.6 are shown in red and more distant similar sequences are shown in orange and blue. **(B)** CDR3 a.a. length of dengue-specific classified antibody sequences. **(C)** Logo-plot of the top 20 dengue-specific classified sequences.

The SVM model could classify broadly neutralizing antibody sequence signatures with 89% prediction accuracy, however this method could not detect these sequence signatures at the repertoire level (Supplementary Figure 6S). Classification performed on the repertoire-level with neural networks was performed using models that have calculated a probability indicating if a repertoire is dengue-challenged or not. However, this approach did not indicate whether a single CDR3 a.a. sequence binds to DENV antigens. We performed training and testing of ML models on dengue-binding antibody sequences. Currently, only few Ab are known to bind dengue (Deng et al., 2013; Hu et al., 2019; Li et al., 2019; Rajamanonmani et al., 2009; Xu et al., 2017). Because these few sequences are not sufficient to train an ML model for sequence classification, we proposed two different approaches that rely both on sequence similarity to generate sequences that could potentially bind dengue antigens and therefore could be used for machine learning (Figure 7). This approach deemed promising as sequence similarity was shown to be an important characteristic for ML to correctly classify antibody sequences. Dengue-neutralizing Ab CDR3 a.a. sequences were collected from previous research (Supplementary Table 3S). A phylogenetic tree was used to detect non-conserved a.a. as a starting point for generating CDR3 a.a. sequences similar to known dengue antibodies. Sequences similar to known dengue-binding antibodies were retrieved in dengue-challenged repertoires. The most similar sequences (coloured sequences) were analyzed through sequence alignment to identify conserved- and non-conserved a.a. All non-conserved a.a. were then replaced by either none or any of the found amino acids for that position and thus generating approximately 1.3 million mutations of the original sequences. Those sequences which were most similar in regards of Levenshtein distance to dengue-binding Ab sequences, and subsequently selected sequences with LD = 1, were retrieved in dengue-challenged repertoires (Supplementary Figure 5S). Non-conserved a.a. were replaced by either none or any of the found amino acids for that position. 141 sequences similar to known dengue-binding antibodies were found in dengue-challenged repertoires. An LSTM model was trained to provide a sequence-level dengue classification. To validate the approach, the known dengue-binding antibodies (*n* = 5), were all classified correctly by the model. Additionally, sequences generated based on mutations of these antibodies were classified with a prediction accuracy of 86.52% and sensitivity 83.36% indicating how many dengue sequences were correctly predicted as dengue specific. By applying the trained ML algorithm on dengue-challenged repertoires, antibodies that could potentially bind to dengue antigens could be selected. A total of approximately 1 million sequences were presented to the model with 34,257 sequences being classified as potential dengue-binding antibody candidates. The 20 highest ranked sequences are shown in Figures 7B,C. As expected, most of the predicted dengue-specific CDR3 a.a. sequences were similar to the m366.6 antibody which was the starting point for simulating the a.a. mutations. These sequences could have also been identified with a sequence similarity network analysis. However, ML also classified sequences which were less similar to the m366.6 antibody. For instance, CDR3 a.a. sequences with a rather large Levenshtein distance to Ab m366.6 (LD = 9, LD = 10) were classified as dengue-binding. This result indicates that ML can classify pontential dengue-binding antibody sequences even if they are not similar to the trained sequences, thus it is a more suitable method for this task compared to similarity networks.

## Discussion

Despite intensive research to understand dengue and the resultant human immune response, as of today neither vaccines nor treatments are available for dengue. The only marketed vaccine rather focuses on dengue subtypes and its efficacy often depends on the clinical history of the patient. Therefore, dengue remains an unresolved thread to global public health, especially in developing countries in South East Asia and Latin America. With advancements in HTS technologies, a multitude of dengue sequence datasets are publicly available and can be used as starting point for training machine learning models.

We have proposed a novel encoding method that takes into consideration the physicochemical properties of each amino acid. By applying a selected set of physicochemical properties to the CDR3 sequence, we established a unique fingerprint for each sequence. Even though our proposed method competes with traditional encoding techniques, we could not demonstrate an added value by applying encoding based on physicochemical rule-based scenarios. Our results indicate that domain-specific knowledge of physicochemical properties of aminoacids is irrelevant for encoding sequence information. Subsequently, we trained different machine learning models to classify CDR3 amino acid sequences first on repertoire and subsequently on the sequence level. By learning intrinsic sequence features from labeled data, the models classified unseen sequences accordingly. Our results demonstrated that one-hot encoding combined with a LSTM ANN architecture led to the highest prediction accuracy of CDR3 sequences. The accuracy achieved was higher than the previous models reaching 71.6% (Shemesh et al., 2021). Similarity networks have potential in mapping and identifying antibody sequences for machine learning training, while these models can outperform similarity analysis in the detection of sequence signatures independently from sequence similarity.

The prediction of repertoire signatures is dependent on the size of the antibody repertoire. Our results indicate that machine learning models perform poorly on small repertoires with less than 1,000 sequences. This might be due to the fact that undersampled repertoires do not reflect properties of the entire repertoire and fail to capture disease-specific characteristics. It requires further research to investigate if there is a general threshold that can be set for the detection of disease-generated patterns in an antibody repertoire.

Our results showed that deep sequencing of the antibody repertoire in dengue infection enables in-depth decoding of dengue-antibody signatures at the repertoire and sequence level. We demonstrated that machine learning can be used to classify CDR3 sequences for DENV repertoire data which represents an important milestone toward the identification of dengue-specific neutralizing antibodies. Further research could apply the proposed architecture on unseen immune repertoire data in order to find sequence-associated signatures of DENV broadly neutralizing antibodies. Therefore, our results show that potential dengue-specific antibody and broadly neutralizing antibody candidates can be generated *de novo* entirely in silico; however, the expression and binding assays are necessary for *in-vitro* validation. The identified broadly neutralizing antibodies could be used to test novel vaccines and design treatments for dengue.

## Data Availability

The original contributions presented in the study are included in the article/Supplementary Material; further inquiries can be directed to the corresponding author.
