# Supplementary material for: Machine Learning Detects Anti-DENV Signatures in Antibody Repertoire Sequences
Source: Front Artif Intell. 2021 Oct 11;4:715462. doi: 10.3389/frai.2021.715462 (PMC8542978; doi:10.3389/frai.2021.715462)
Supplement: Supplementary file 1 [file DataSheet2.pdf]

Machine learning detects anti-DENV signatures in antibody repertoire sequences

Alexander Horst[1], Erand Smakaj[1], Eriberto Noel Natali[1], Deniz Tosoni[1], Lmar Marie Babrak[1], Patrick Meier[1], Enkelejda Miho[1,2,3]

[1]FHNW University of Applied Sciences and Arts Northwestern Switzerland, School of Life Sciences, Muttenz, Switzerland
[2]SIB Swiss Institute of Bioinformatics, Lausanne, Switzerland
[3]aiNET GmbH, Basel, Switzerland

SUPPLEMENTARY INFORMATION

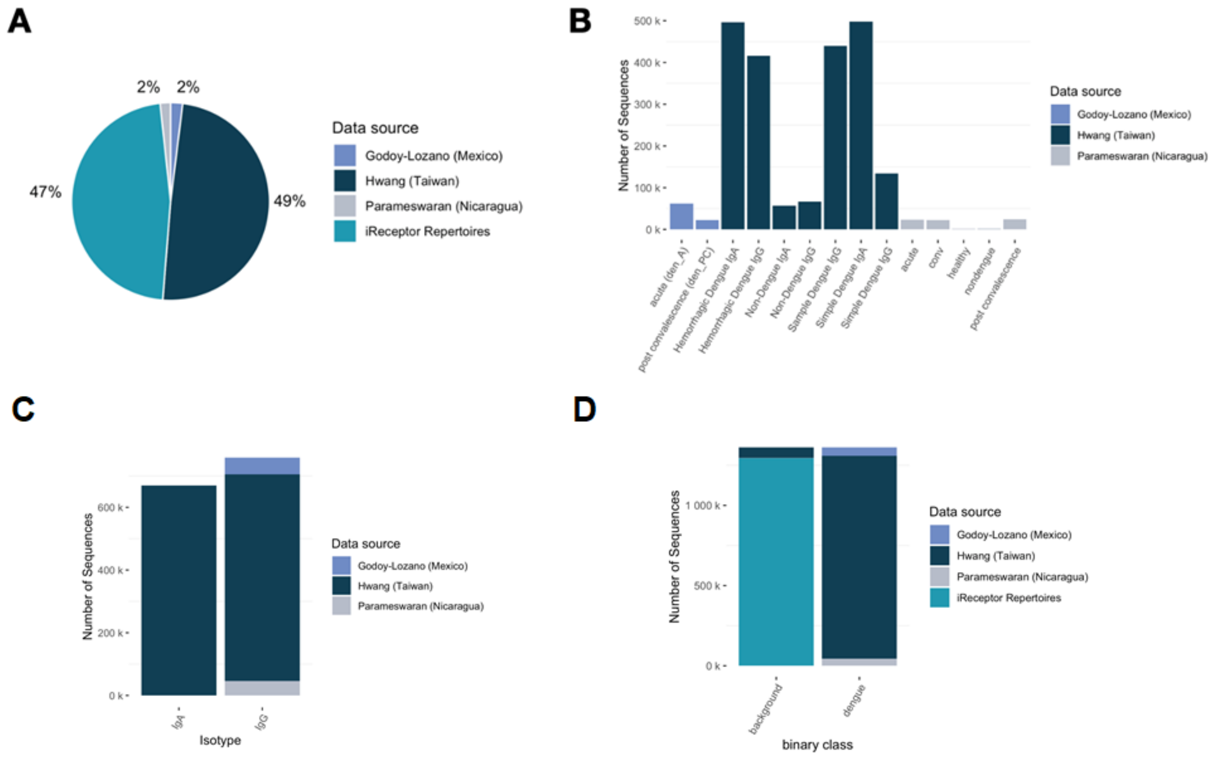

Figure 1S. Overview of analyzed dengue antibody repertoires. (A) Source overview of the data. The majority (49%) of the dengue data were found in Huang et al. from Taiwanese dengue samples while Parameswaren (Niceraguan dengue samples) data and Godoy-Lozano (Mexican dengue samples) contributed with 2% each. 47% of the data were collected from non-dengue challenged repertoires found on iReceptor. (B) The datasets used originally came with a multitude of classes leading to a total of 14 different classes which were mapped into two classes (C): dengue / non-dengue. (D) Classes are composed of heavy chain antibody sequences. (E) Flowchart of data processing and analysis.

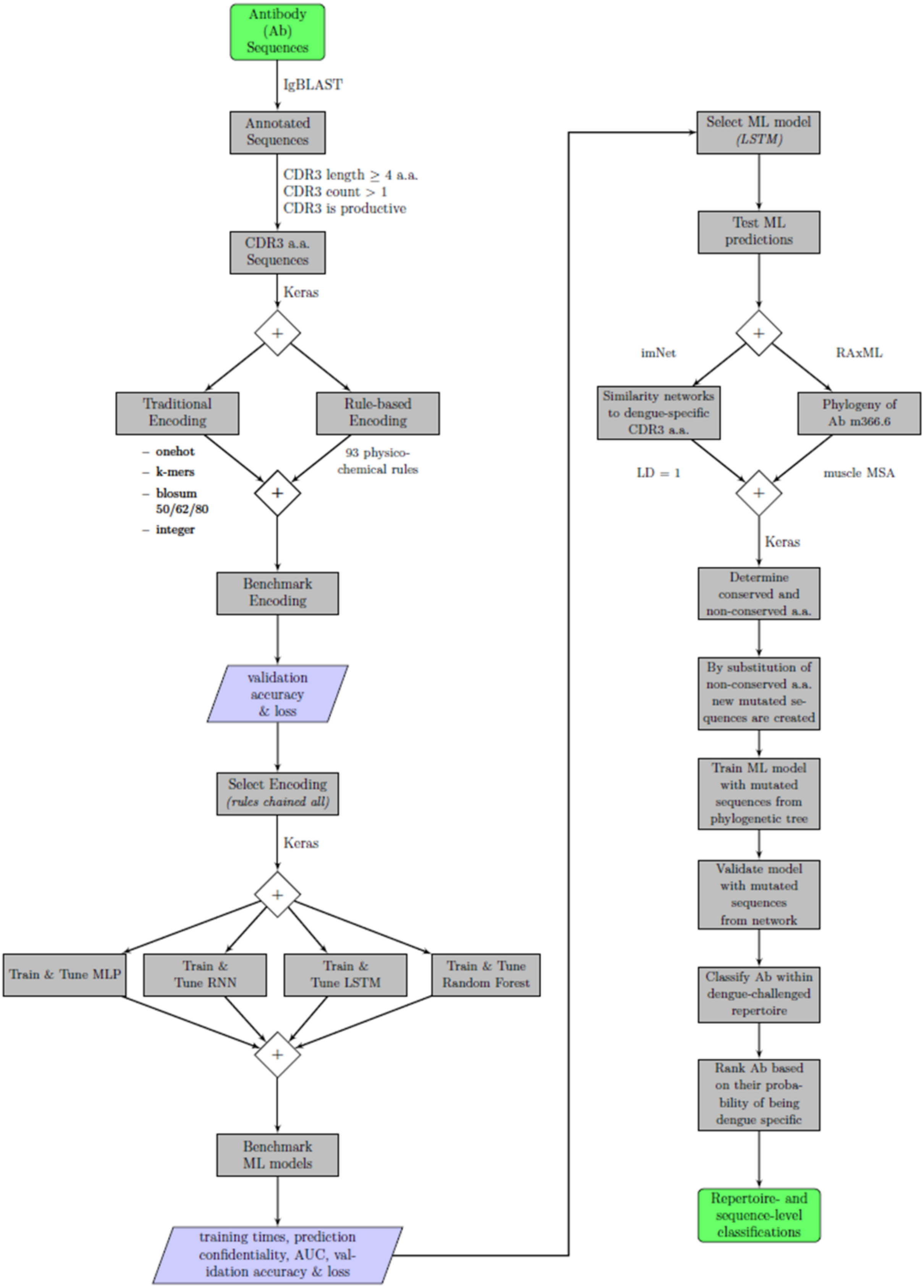

Figure 2S. Flowchart of data processing and analysis.

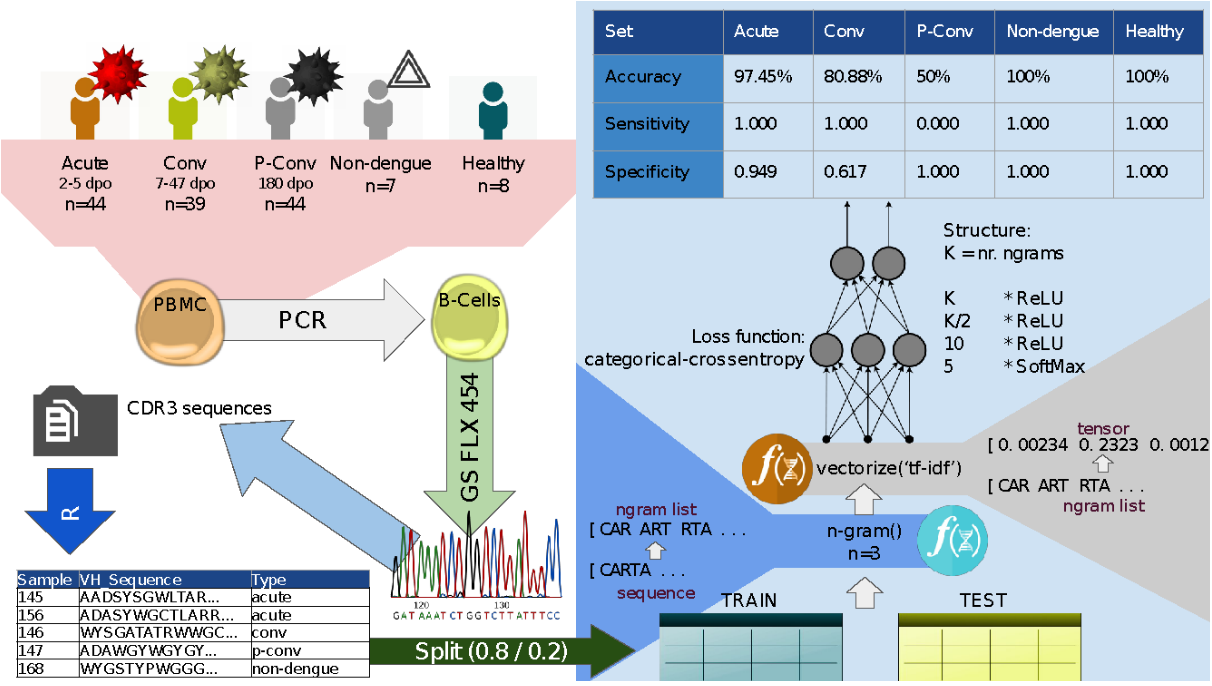

Figure 3S. CDR3 sequences were transformed into series of trigrams after and the resulting vectors were transformed to tensors using tf-idf function. The resulting tensors are used to train a deep learning network to classify each CDR3 with one of the given labels. Once the model was trained, the class of each sequence of a given repertoire was predicted. The repertoire label was then assigned depending on a majority vote for the sequence labels it contained. The model can predict with an accuracy of more than 80% all sets with the original label except for the post convalescent (P-Conv) repertoires. P-Conv was generally mapped to Conv and sometimes to Acute.

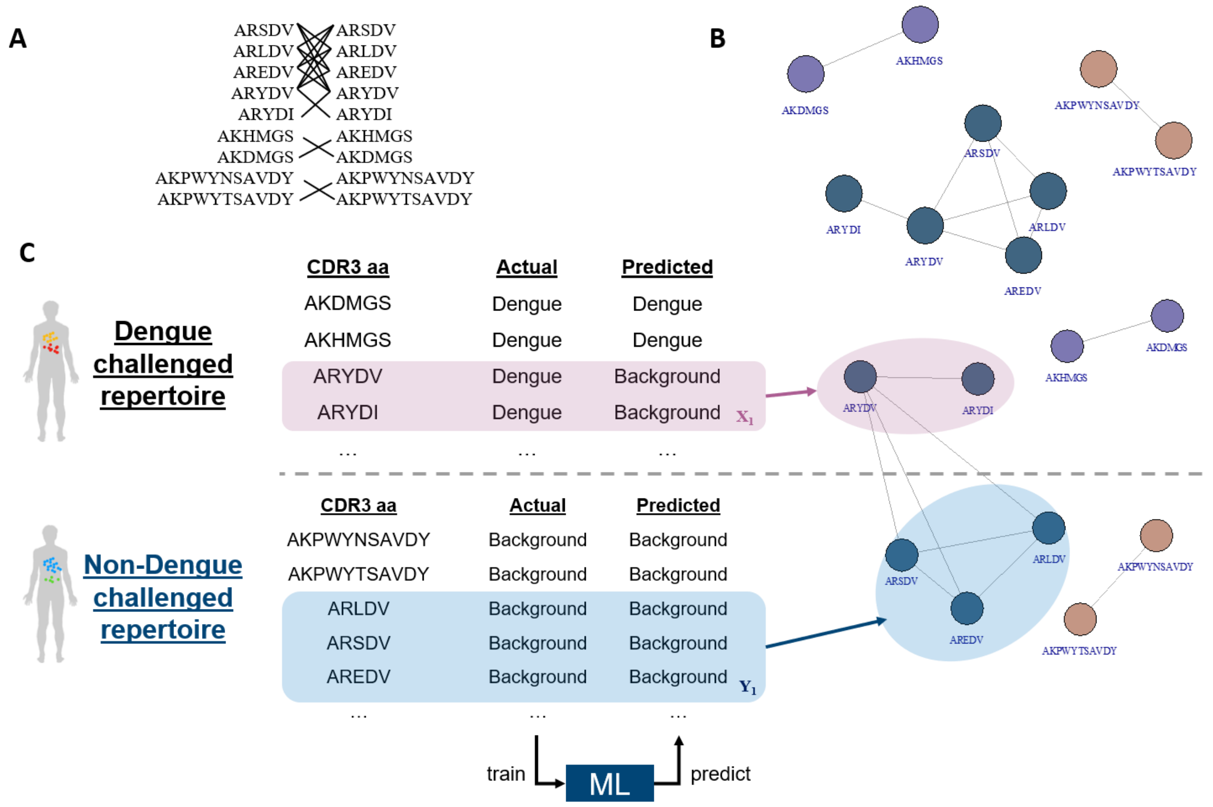

Figure 4S. Similarity networks of dengue-challenged and background sequences. (A) For each CDR3 amino acid sequence, its most similar sequences with Levenshtein distance (LD) = 1, change of one amino acid, were derived. (B) Similar CDR3 can then be represented as a similarity network. Each node represents a single sequence and is connected to other sequences within the group for LD=1. (C) Each repertoire contained CDR amino acid sequences, which were all equally dengue or background labelled. These were fed into the ML model. Some sequences within repertoire 1 (x_1) were misclassified. Comparing these sequences with the similarity network of (B), indicates that these sequences are highly similar to sequences found in repertoire 2 (y_1) and therefore, classified equally to those sequences.

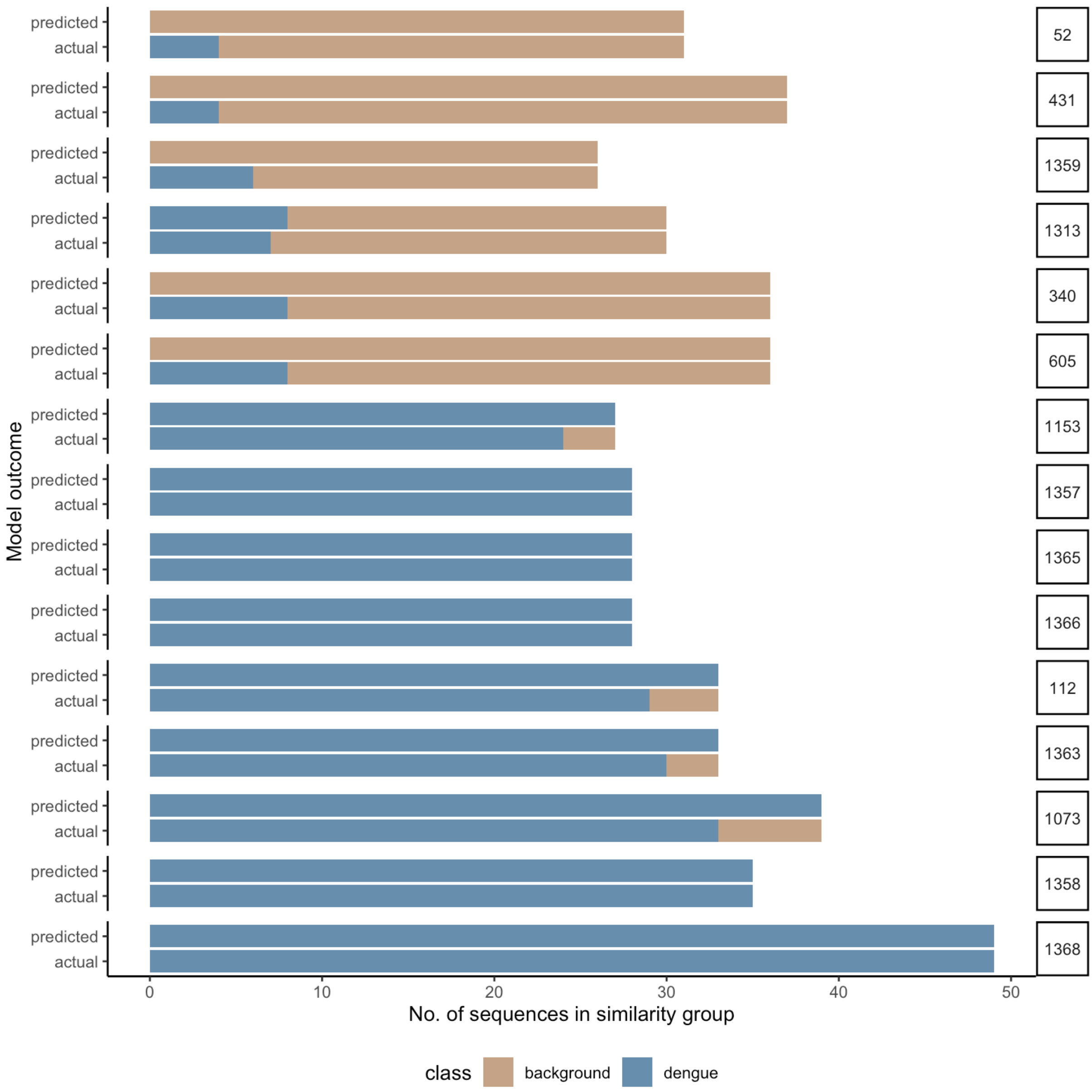

Figure 5S. In each similarity network the actual class is opposed to the predicted class. For readability, the figure shows only similarity networks with a minimum of 10% dengue sequences.

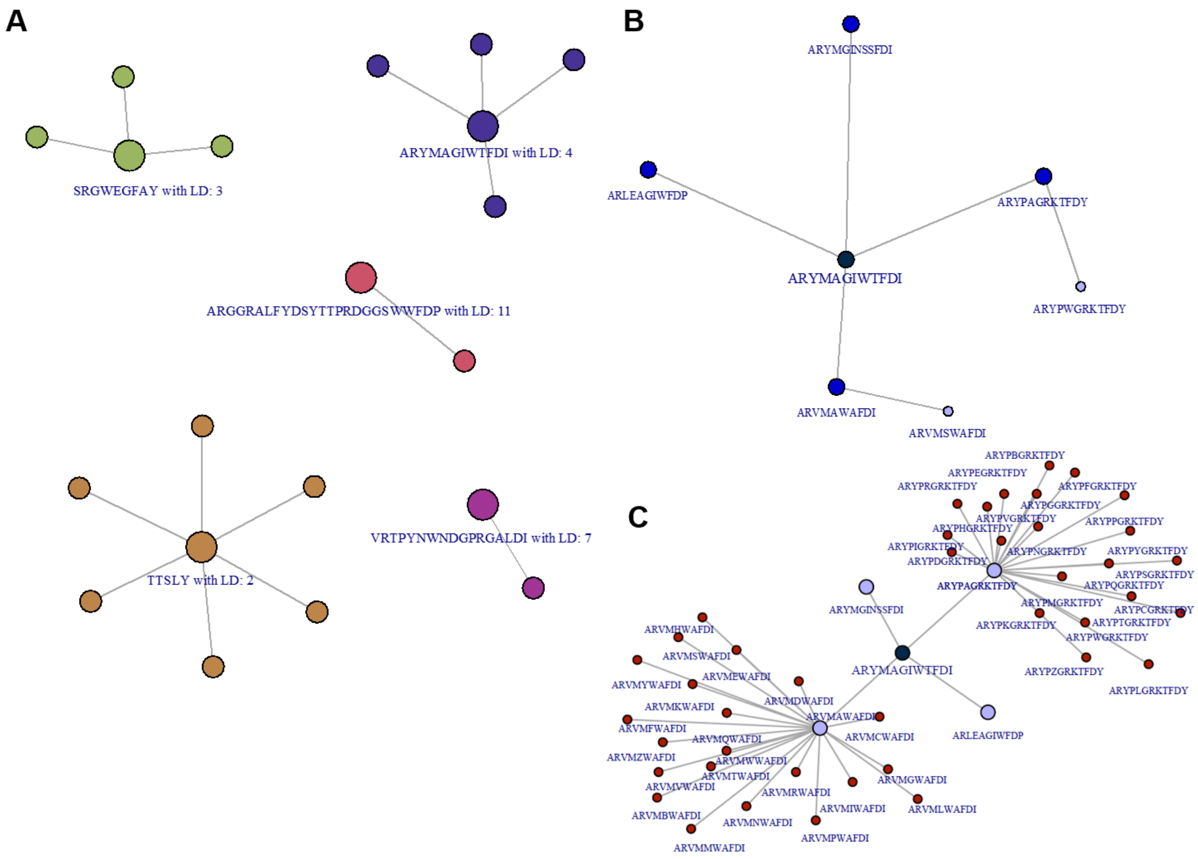

Figure 6S. Similarity networks of dengue-specific Ab CDR3 sequences mapped to dengue-challenged repertoires. (A) Similar sequences within dengue-challenged repertoires to known dengue Ab. (B) Similar to (A) with LD = 1. (C) Mutations were generated by aligning sequences found in (B) and substitution of non-conserved a.a.

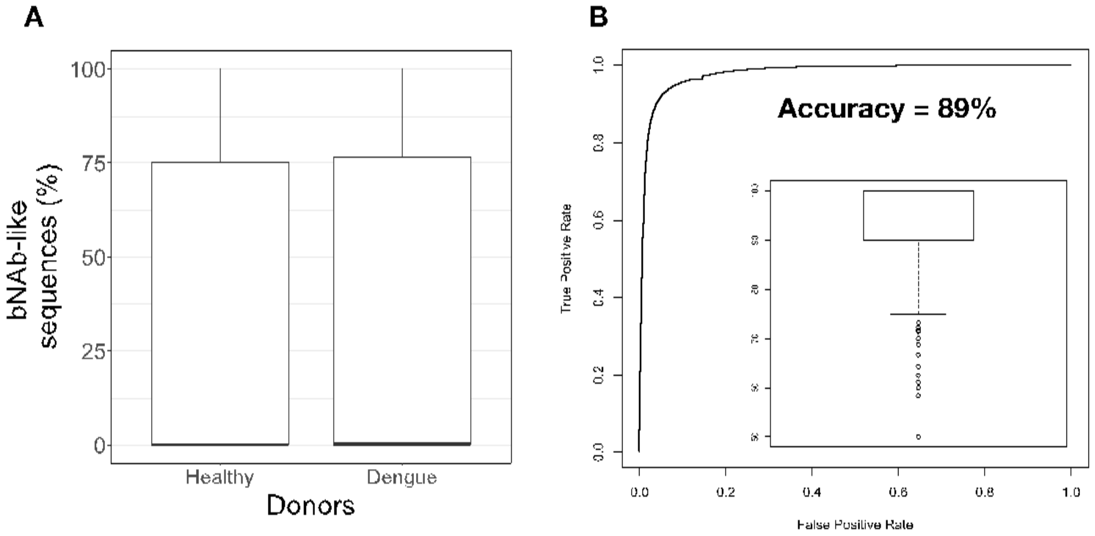

Figure 7S. SVM method detects bNAb-like sequence features. (A) Detection of dengue-bNAb sequence signatures at the antibody repertoire level. (B) Prediction of bNAb-like sequence features.

Hyperparameter	Values explored	Details
Epochs	20	Number of training epochs avoided overfitting by adding call-back function
Hidden Layer	1, 2, 3, 5, 10	Starting from universal approximation theorem (one single hidden layer) up to five hidden layers
Number of neurons	32, 64, 128, 256, 512	Number of neurons per hidden layer
Activation function	ReLU, Sigmoid	ReLU activation for hidden layer, Sigmoid for output layer
Loss function	Binary crossentropy	Probability for a binary classification problem
Dropout	0%, 10%, 20%, 30%, 50%	Dropout rate for nodes that are being randomly skipped during training
Classification Problem	Binary	Dengue / Background (may be subject of change)

Table 1S. Hyper-parameters used for tuning artificial neural networks.

Hyperparameter	Values explored	Details
Ntrees	300	Number of trees the random forest consists of
Ntry	14, 27, 54	Number of splits at each node

Table 2S. Hyperparameters used for training the random forest

Name	Specificity	CDR3 a.a. VH	Reference
M366.6	DENV1-4	ARYMAGIWTFDI	Hu et al., 2019
M360.6	DENV1-4	VRTPYNWNDGPRGALDI	Hu et al., 2019
SIgN-3C	DENV1-4	ARGGRALFYDSYTTPRDGGSWWFDP	Xu et al., 2017
9F12	DENV1-4	TTSLY	Rajamanonmani et al., 2009

AJ131288	DENV1	SRGWEGFAY	Thullier et al., 1999

Table 3S. Neutralizing dengue Ab CDR3 a.a. sequences

bNAb name	Reference
9F12	(42)
3E31	(43)
M366.6	(44)
1A1-D2	(45)
M360.6	(44)
2H12	(46)
4E11	(47)
4E5A	(48)
Ab513	(49)
SIgN-3C	(50)
2A10G6	(51)
1C19	(52)
1N5	(52)
1M7	(52)
d448	(53)
J8	(54)

**J9**	**(54)**
**EDE1 C10**	**(55)**
**EDE1 C8**	**(55)**
**EDE2 A11**	**(55)**
**EDE2 B7**	**(55)**
**D23-1B3B9**	**(56)**
**DVD-1A1D-2A10**	**(57)**
**1F4**	**(58)**
**14C10**	**(59)**
**2D22**	**(60)**

Table 4S. Collected dengue-specific bNAbs names and literature references

Publication	Dataset	Raw reads
Godoy Lozano et al.	SRR2925333	30834
	SRR2925334	39239
	SRR2925335	38722
	SRR2925336	28965
	SRR2925337	37407

	SRR2925338	42552
	SRR2925339	53555
	SRR2925340	62564
	SRR2925341	34823
	SRR2925677	66787
	SRR2925678	65871
	SRR2925679	57342
	SRR2925680	54776
	SRR2925681	60130
	SRR2925682	61684
	SRR2925683	42482
	SRR2925684	118864
	SRR2925685	79255
	SRR2925686	84812
	SRR2925687	57857
	SRR2925689	134520
	SRR2925690	77428
	SRR2925692	73251

	SRR2925693	75976
	SRR2925694	116670
	SRR2925695	113409
	SRR2925696	103995
	SRR2925698	78178
	SRR2925699	75040
	SRR2925701	80783
Parameswaran et al.	Acute_Bulk_148_acute_Bulk	2260
	Acute_Bulk_172_Acute_Bulk	8096
	Acute_Bulk_194_Acute_Bulk	4135
	Acute_Bulk_199_Acute_Bulk	13138
	Acute_Bulk_203_Acute_Bulk	8268
	Acute_Bulk_208_Acute_Bulk	3757
	Acute_Bulk_232_Acute_Bulk	8377
	Acute_Bulk_237_Acute_Bulk	8935
	Acute_Bulk_238_Acute_Bulk	10734
	Acute_Bulk_240_Acute_Bulk	9273
	Acute_Bulk_249_Acute_Bulk	8781

	Acute_Bulk_252_Acute_Bulk	4682
	Acute_Bulk_255_Acute_Bulk	3678
	Acute_Bulk_275_Acute_Bulk	3270
	Acute_Bulk_276_Acute_Bulk	4042
	Acute_Bulk_287_Acute_Bulk	4556
	Acute_Bulk_289_Acute_Bulk	6184
	Acute_Bulk_299_Acute_Bulk	5682
	Acute_Bulk_301_Acute_Bulk	11785
	Acute_Bulk_307_Acute_Bulk	4791
	Acute_Bulk_311_Acute_Bulk	7818
	Acute_Bulk_320_Acute_Bulk	4590
	Acute_Bulk_346_Acute_Bulk	17
	Acute_Bulk_376_Acute_Bulk	2587
	Acute_Bulk_391_Acute_Bulk	3825
	Acute_Bulk_422_Acute_Bulk	1885
	Acute_Bulk_444_Acute_Bulk	4286
	Acute_Bulk_455_Acute_Bulk	4922
	Acute_Bulk_479_Acute_Bulk	4207

	Acute_Bulk_481_Acute_Bulk	3636
	Acute_Bulk_489_Acute_Bulk	4484
	Acute_Bulk_500_Acute_Bulk	4588
	Acute_Bulk_514_Acute_Bulk	3537
	Acute_Bulk_515_Acute_Bulk	4128
	Acute_Bulk_517_Acute_Bulk	3441
	Acute_Bulk_520_Acute_Bulk	8775
	Acute_Bulk_524_Acute_Bulk	3951
	Acute_Bulk_529_Acute_Bulk	9483
	Acute_Bulk_543_Acute_Bulk	3748
	Acute_Bulk_551_Acute_Bulk	1912
	Acute_Bulk_555_Acute_Bulk	9638
	Acute_Bulk_558_Acute_Bulk	2687
	Acute_Bulk_563_Acute_Bulk	4686
	Acute_Bulk_569_Acute_Bulk	2683
	Acute_IGHA_172_Acute_IGHA	3
	Acute_IGHA_194_Acute_IGHA	6
	Acute_IGHA_199_Acute_IGHA	12

	Acute_IGHA_208_Acute_IGHA	7
	Acute_IGHA_232_Acute_IGHA	7
	Acute_IGHA_249_Acute_IGHA	9
	Acute_IGHA_255_Acute_IGHA	3
	Acute_IGHA_289_Acute_IGHA	3
	Acute_IGHA_301_Acute_IGHA	8
	Acute_IGHA_376_Acute_IGHA	2
	Acute_IGHA_479_Acute_IGHA	5
	Acute_IGHD_148_acute_IGHD	51
	Acute_IGHD_232_Acute_IGHD	173
	Acute_IGHD_287_Acute_IGHD	70
	Acute_IGHD_307_Acute_IGHD	84
	Acute_IGHD_489_Acute_IGHD	127
	Acute_IGHD_563_Acute_IGHD	144
	Acute_IGHG_172_Acute_IGHG	2
	Acute_IGHG_199_Acute_IGHG	5
	Conv_Bulk_148_Conv_Bulk	6383
	Conv_Bulk_172_Conv_Bulk	10511

	Conv_Bulk_194_Conv_Bulk	12021
	Conv_Bulk_199_Conv_Bulk	9442
	Conv_Bulk_203_Conv_Bulk	14125
	Conv_Bulk_208_Conv_Bulk	8564
	Conv_Bulk_232_Conv_Bulk	6263
	Conv_Bulk_237_Conv_Bulk	8924
	Conv_Bulk_238_Conv_Bulk	7642
	Conv_Bulk_240_Conv_Bulk	10152
	Conv_Bulk_249_Conv_Bulk	8394
	Conv_Bulk_252_Conv_Bulk	3359
	Conv_Bulk_255_Conv_Bulk	4548
	Conv_Bulk_265_Conv_Bulk	8407
	Conv_Bulk_275_Conv_Bulk	3809
	Conv_Bulk_276_Conv_Bulk	4917
	Conv_Bulk_287_Conv_Bulk	5538
	Conv_Bulk_289_Conv_Bulk	5642
	Conv_Bulk_299_Conv_Bulk	5151
	Conv_Bulk_301_Conv_Bulk	7172

	Conv_Bulk_376_Conv_Bulk	3224
	Conv_Bulk_422_Conv_Bulk	1965
	Conv_Bulk_444_Conv_Bulk	4833
	Conv_Bulk_455_Conv_Bulk	4687
	Conv_Bulk_479_Conv_Bulk	4421
	Conv_Bulk_481_Conv_Bulk	3929
	Conv_Bulk_489_Conv_Bulk	5404
	Conv_Bulk_500_Conv_Bulk	3972
	Conv_Bulk_514_Conv_Bulk	3795
	Conv_Bulk_515_Conv_Bulk	3440
	Conv_Bulk_517_Conv_Bulk	3782
	Conv_Bulk_520_Conv_Bulk	2965
	Conv_Bulk_524_Conv_Bulk	3122
	Conv_Bulk_529_Conv_Bulk	3434
	Conv_Bulk_543_Conv_Bulk	3676
	Conv_Bulk_551_Conv_Bulk	2154
	Conv_Bulk_555_Conv_Bulk	3980
	Conv_Bulk_563_Conv_Bulk	5445

	Conv_Bulk_569_Conv_Bulk	4617
	Conv_IGHA_199_Conv_IGHA	9
	Conv_IGHA_203_Conv_IGHA	8
	Conv_IGHA_232_Conv_IGHA	9
	Conv_IGHA_238_Conv_IGHA	5
	Conv_IGHA_240_Conv_IGHA	13
	Conv_IGHA_249_Conv_IGHA	5
	Conv_IGHA_252_Conv_IGHA	2
	Conv_IGHA_265_Conv_IGHA	6
	Conv_IGHA_287_Conv_IGHA	3
	Conv_IGHA_289_Conv_IGHA	5
	Conv_IGHA_299_Conv_IGHA	5
	Conv_IGHA_444_Conv_IGHA	7
	Conv_IGHD_172_Conv_IGHD	143
	Conv_IGHD_203_Conv_IGHD	172
	Conv_IGHD_237_Conv_IGHD	234
	Conv_IGHD_265_Conv_IGHD	268
	Conv_IGHD_287_Conv_IGHD	86

	Conv_IGHD_289_Conv_IGHD	120
	Conv_IGHD_299_Conv_IGHD	72
	Conv_IGHD_444_Conv_IGHD	151
	Conv_IGHD_455_Conv_IGHD	129
	Conv_IGHD_489_Conv_IGHD	144
	Conv_IGHD_515_Conv_IGHD	143
	Conv_IGHD_517_Conv_IGHD	174
	Conv_IGHD_524_Conv_IGHD	61
	Conv_IGHD_555_Conv_IGHD	109
	Conv_IGHD_569_Conv_IGHD	150
	healthy_Bulk_1503_healthy_Bulk	2627
	healthy_Bulk_223_healthy_Bulk	1993
	healthy_Bulk_2603_healthy_Bulk	2514
	healthy_Bulk_331_healthy_Bulk	622
	healthy_Bulk_4057_healthy_Bulk	2862
	healthy_Bulk_4431_healthy_Bulk	2424
	healthy_Bulk_4468_healthy_Bulk	2794
	healthy_Bulk_4873_healthy_Bulk	1563

	healthy_IGHD_331_healthy_IGHD	25
	healthy_IGHD_4057_healthy_IGHD	159
	nonDengue_Bulk_171_nonDengue_Bulk	3055
	nonDengue_Bulk_207_nonDengue_Bulk	4014
	nonDengue_Bulk_209_nonDengue_Bulk	3570
	nonDengue_Bulk_220_nonDengue_Bulk	3211
	nonDengue_Bulk_330_nonDengue_Bulk	1856
	nonDengue_Bulk_332_nonDengue_Bulk	1774
	nonDengue_Bulk_345_nonDengue_Bulk	1885
	nonDengue_IGHD_332_nonDengue_IGHD	74
	PConv_Bulk_148_PConv_Bulk	6019
	PConv_Bulk_172_PConv_Bulk	11942
	PConv_Bulk_194_PConv_Bulk	11751
	PConv_Bulk_199_PConv_Bulk	9083

	PConv_Bulk_203_PConv_Bulk	15395
	PConv_Bulk_208_PConv_Bulk	8914
	PConv_Bulk_232_PConv_Bulk	7091
	PConv_Bulk_237_PConv_Bulk	9618
	PConv_Bulk_238_PConv_Bulk	7022
	PConv_Bulk_240_PConv_Bulk	10648
	PConv_Bulk_249_PConv_Bulk	7334
	PConv_Bulk_252_PConv_Bulk	3552
	PConv_Bulk_255_PConv_Bulk	4688
	PConv_Bulk_265_PConv_Bulk	11107
	PConv_Bulk_275_PConv_Bulk	3751
	PConv_Bulk_276_PConv_Bulk	4971
	PConv_Bulk_287_PConv_Bulk	5134
	PConv_Bulk_289_PConv_Bulk	5824
	PConv_Bulk_299_PConv_Bulk	4732
	PConv_Bulk_301_PConv_Bulk	7141
	PConv_Bulk_307_PConv_Bulk	4894
	PConv_Bulk_311_PConv_Bulk	1129

	PConv_Bulk_320_PConv_Bulk	2663
	PConv_Bulk_376_PConv_Bulk	3309
	PConv_Bulk_391_PConv_Bulk	3960
	PConv_Bulk_422_PConv_Bulk	3688
	PConv_Bulk_444_PConv_Bulk	4370
	PConv_Bulk_455_PConv_Bulk	3984
	PConv_Bulk_479_PConv_Bulk	6130
	PConv_Bulk_481_PConv_Bulk	3731
	PConv_Bulk_489_PConv_Bulk	3947
	PConv_Bulk_500_PConv_Bulk	4709
	PConv_Bulk_514_PConv_Bulk	4261
	PConv_Bulk_515_PConv_Bulk	5451
	PConv_Bulk_517_PConv_Bulk	3465
	PConv_Bulk_520_PConv_Bulk	3469
	PConv_Bulk_524_PConv_Bulk	3535
	PConv_Bulk_529_PConv_Bulk	2172
	PConv_Bulk_543_PConv_Bulk	3578
	PConv_Bulk_551_PConv_Bulk	2162

	PConv_Bulk_555_PConv_Bulk	3647
	PConv_Bulk_558_PConv_Bulk	3542
	PConv_Bulk_563_PConv_Bulk	4416
	PConv_Bulk_569_PConv_Bulk	3893
	PConv_IGHA_203_PConv_IGHA	11
	PConv_IGHA_208_PConv_IGHA	6
	PConv_IGHA_238_PConv_IGHA	6
	PConv_IGHA_249_PConv_IGHA	8
	PConv_IGHA_265_PConv_IGHA	4
	PConv_IGHA_275_PConv_IGHA	3
	PConv_IGHA_276_PConv_IGHA	5
	PConv_IGHA_287_PConv_IGHA	3
	PConv_IGHA_289_PConv_IGHA	5
	PConv_IGHA_301_PConv_IGHA	2
	PConv_IGHA_320_PConv_IGHA	5
	PConv_IGHA_391_PConv_IGHA	5
	PConv_IGHA_444_PConv_IGHA	3
	PConv_IGHA_515_PConv_IGHA	3

	PConv_IGHD_203_PConv_IGHD	152
	PConv_IGHD_240_PConv_IGHD	241
	PConv_IGHD_289_PConv_IGHD	109
	PConv_IGHD_376_PConv_IGHD	72
	PConv_IGHD_481_PConv_IGHD	106
	PConv_IGHD_514_PConv_IGHD	105
	PConv_IGHD_520_PConv_IGHD	69
	PConv_IGHD_543_PConv_IGHD	76
	PConv_IGHE_569_PConv_IGHE	2
	PConv_IGHG_563_PConv_IGHG	2
	PConv_IGHM_563_PConv_IGHM	4
Huang et al.	ERR1391650_2	1238811
	ERR1391651_2	2036920
	ERR1391652_2	1066006
	ERR1391653_2	679449
	ERR1391654_2	1046009
	ERR1391655_2	1184586
	ERR1391656_2	1408308

	ERR1391657_2	1188253
	ERR1391658_2	1964663
	ERR1391659_2	4709751
	ERR1391660_2	1757845
	ERR1391661_2	2566743
	ERR1391662_2	1718620
	ERR1391664_2	1585455
	ERR1391665_2	1984064
	ERR1391666_2	595102
	ERR1391667_2	732632
	ERR1391668_2	576842
	ERR1391669_2	425768
	ERR1391670_2	569966
	ERR1391671_2	1001074
	ERR1391672_2	2762664
	ERR1391673_2	533375
	ERR1391674_2	1079853
	ERR1391675_2	1845679

	ERR1391676_2	929460
	ERR1391677_2	1988965
	ERR1391678_2	1665303
	ERR1391679_2	1946138
	ERR1391680_2	1042860
	ERR1391681_2	1164252
	ERR1391682_2	641950
	ERR1391683_2	1034967
	ERR1391684_2	1463226
	ERR1391685_2	1143995
	ERR1391686_2	1497531
	ERR1391687_2	1632310
	ERR1391688_2	931297
	ERR1391689_2	1028143
	ERR1391690_2	515190
	ERR1391691_2	1033327
	ERR1391692_2	2499540
	ERR1391693_2	2937510

	ERR1391694_2	1721941
	ERR1391695_2	1659859
	ERR1391696_2	766026
	ERR1391697_2	1184785
	ERR1391698_2	1684683
	ERR1391699_2	1024602
	ERR1391700_2	646467
	ERR1391701_2	888643
	ERR1391702_2	505264
	ERR1391703_2	948047
	ERR1391704_2	2167948
	ERR1391705_2	1705755
	ERR1391706_2	3208964
	ERR1391707_2	2797468
	ERR1391708_2	1829050
	ERR1391709_2	2565672

Table 5S. Reference, dataset name and raw reads per dataset used in this study.
