# Supplementary material for: Machine Learning Detects Anti-DENV Signatures in Antibody Repertoire Sequences
Source: Front Artif Intell. 2021 Oct 11;4:715462. doi: 10.3389/frai.2021.715462 (PMC8542978; doi:10.3389/frai.2021.715462)
Supplement: Supplementary file 2 [file DataSheet1.PDF]

Appendix 1

Rule library

The following table provides an overview of the physicochemical properties used for the rule-based encoding.

**Rule**	**Property**	**Value**	**Amino acid**
1	Hydropathy	neutral	GHPSTY
2		hydrophobic	ACFILMVW
3		hydophilic	DEKNQR
4	Volume (in Å3)	Very small [60-90] & Small [108-117]	CDFGNPSTVW
5		Medium [138-154]	EHQV
6		Large [162-174] and Very large [189-228]	FIKLMRWY
7	Chemical	aliphatic	AGILPV
8		aromatic	FWY
9		sulfur	CM
10		hydroxyl	ST
11		basic	HKR
12		acidic	DE
13		amide	NQ
14	Charge	positive	HKR
15		negative	DE
16		no charge	ACFGILMNPQSTVWY

17		positive or negative charnge	DEHRK
18		polar	CNQST
19		Polar/uncharged	CNQSTY
20		polar/charged	HKR
21		Amphipathic (found on the surface of proteins)	MWY
22	Hydrogen donor or acceptor atoms	donor	KRW
23		acceptor	DE
24		donor and acceptor	HNQSTY
25		none	ACFGILMPVW
26	Flexibility	Flexibility was scored by Huang, 2003 from 1-10; AA with score of 1-5=flexible (+1) and 6-10 = not flexible (0)	ADEFGLNQST
27		Atomic displacement factors/B-factors (Smith, 2003) Flexible +1; Rigid (0)	ACHILMVWY
28	Essentiality	essential aa (body can't make) (+1); nonessential (body can make)(-1)	FHIKLMTVW
29	pKa1= -carboxyl group	Less than pka1=2.1 (+1); greather than pka1=2.1 = 0 (2.1 is aproximately the median and mean)	CDFHNPTW

30	pKa2 = -ammonium ion	Less than pka2=9.2 (+1); greather than pka2=9.2 (0) (9.2 is aproximately the median)	CFHKNQRSTY
31	pKa3 = side chain group	Less than 10 (+1); greater than 10 (0)	DEH
32	pI	Less than 5.9 (+1); Greater an 5.9 (0)	CDEFMNQSTWY
33	Percent frequncy in vertebrates	Less than 4.9 (0); greater than 4.9 (+1)	GADGKLPSTV
34	Secondary Structure	Free energy: alpha sheets propensity kcal/mol (higher number = less likely alpha helix); Greater than 0.55 (0), less than 0.55 (+1) (Pace, 1998)	AEFIKLMQRSTVW
35		Free energy:alpha sheets propensity kJ/mol (higher number = less likely alpha helix) (+1 for less than 2.5; 0 for greater than 2.5)	AEIKLMQRSWY
37	Secondary Structure: propensity of location (Fujiwara, 2012)	alpha-helix favored	AEKLQ
38		alpha-helix breaker	CDFGNPSTVW
39		alpha-helix neutral	DIMY
40	alpha-helix buried residues	alpha-helix breaker	CDGHNPSTV
41		-helix-favored amino acid	AEKLMQR

42		neutral amino acid	FIWY
43	alpha-helix total residues	-helix breaker	CDGHNPST
44		-helix-favored amino acid	AEKLMQR
45		neutral amino acid	FIVWY
46	beta-strand exposed residues	beta-strand breaker	ADEGNPS
47		beta-strand-favored amino acid	CFHIKLRTVWY
48		neutral amino acid	MQ
49	beta-strand buried residues	beta-strand breaker	ADEGMNPQRS
50		beta-strand-favored amino acid	IVY
51		neutral amino acid	CFHKLTW
52	beta-strand total residues	beta-strand breaker	ADEGKNPQRS
53		beta-strand-favored amino acid	CFILTVWY
54		neutral amino acid	HM
55		Non-polar	GAVCPLIMWF
56	Single amino acids	Alanine	A
57		Cystine	C
58		Aspartic acid	D
59		Glutamic acid	E
60		Phenylalanine	F
61		Glycine	G
62		Histidine	H
63		Isoleucine	I
64		Lysine	K
65		Leucine	L
66		Methionine	M

67		Asparagine	N
68		Proline	P
69		Glutarnine	Q
70		Arginine	R
71		Serine	S
72		Threonine	T
73		Valine	V
74		Tryptophan	W
75		Tyrosine	Y
76	Repeated amino acids	Amino acids repeated once	-
77		Amino acids repeated three times	-
78		Amino acids repeated four times	-
79		Alpha helix propensity of variable region, 1 for amino acids with propensity 1	AEFKLMQRW
80	Propensity	Beta sheet propensity of variable region, 1 for amino acids with propensity 1	CFHILTVWY
81		Loop propensity of variable region, 1 for amino acids with propensity 1	DGHNPS
82		Hinge propensity of variable region, 1 for amino acids with propensity 1	AKNPQSTY

83	Solubility	Amino acids highly soluble (S100g/100ml)	CKPT
84		Amino acids intermediately soluble (10S100g/100ml)	AGR
85		Amino acids poorly soluble (S10g/100ml)	DEHILMNQSVWY
86	Molecular weight	Amino acids with low MW (100)	AG
87		Amino acids with intermediate MW (100MW170)	CDEFHIKLMNPQSTV
88		Amino acids with high MW (MW170)	RWY
89		Amino acids with N groups on side chain	HKNPQRW
90		Branched-chain aminoacids (BCAA)	ILV
91	Number of possible hydrogen bonds	Amino acids that can make 4 H bonds	DEHNQRST
92		Amino acids that can make 3 H bonds	WY
93		Amino acids that can make 2 H bonds	ACFGIKLMPV
